# Pharmacokinetic-Pharmacodynamic Target Attainment Analyses Evaluating Omadacycline Dosing Regimens for the Treatment of Patients with Community-Acquired Bacterial Pneumonia Arising from Streptococcus pneumoniae and Haemophilus influenzae

**DOI:** 10.1128/aac.02213-21

**Published:** 2023-03-22

**Authors:** Sujata M. Bhavnani, Jeffrey P. Hammel, Elizabeth A. Lakota, Michael Trang, Justin C. Bader, Catharine C. Bulik, Brian D. VanScoy, Christopher M. Rubino, Michael D. Huband, Lawrence Friedrich, Judith N. Steenbergen, Paul G. Ambrose

**Affiliations:** a Institute for Clinical Pharmacodynamics, Inc., Schenectady, New York, USA; b JMI Laboratories, North Liberty, Iowa, USA; c Paratek Pharmaceuticals, Inc., King of Prussia, Pennsylvania, USA

**Keywords:** omadacycline, pharmacokinetics-pharmacodynamics, community-acquired bacterial pneumonia, *Streptococcus pneumoniae*, *Haemophilus influenzae*, simulations

## Abstract

Omadacycline, a novel aminomethylcycline with *in vitro* activity against Gram-positive and -negative organisms, including Streptococcus pneumoniae and Haemophilus influenzae, is approved in the United States to treat patients with community-acquired bacterial pneumonia (CABP). Using nonclinical pharmacokinetic-pharmacodynamic (PK-PD) targets for efficacy and *in vitro* surveillance data for omadacycline against S. pneumoniae and H. influenzae, and a population pharmacokinetic model, PK-PD target attainment analyses were undertaken using total-drug epithelial lining fluid (ELF) and free-drug plasma exposures to evaluate omadacycline 100 mg intravenously (i.v.) every 12 h or 200 mg i.v. every 24 h (q24h) on day 1, followed by 100 mg i.v. q24h on day 2 and 300 mg orally q24h on days 3 to 5 for patients with CABP. Percent probabilities of PK-PD target attainment on days 1 and 2 by MIC were assessed using the following four approaches for selecting PK-PD targets: (i) median, (ii) second highest, (iii) highest, and (iv) randomly assigned total-drug ELF and free-drug plasma ratio of the area under the concentration-time curve to the MIC (AUC/MIC ratio) targets associated with a 1-log_10_ CFU reduction from baseline. Percent probabilities of PK-PD target attainment based on total-drug ELF AUC/MIC ratio targets on days 1 and 2 were ≥91.1% for S. pneumoniae for all approaches but the highest target and ≥99.2% for H. influenzae for all approaches at MIC_90_s (0.12 and 1 μg/mL for S. pneumoniae and H. influenzae, respectively). Lower percent probabilities of PK-PD target attainment based on free-drug plasma AUC/MIC ratio targets were observed for randomly assigned and the highest free-drug plasma targets for S. pneumoniae and for all targets for H. influenzae. These data provided support for approved omadacycline dosing regimens to treat patients with CABP and decisions for the interpretive criteria for the *in vitro* susceptibility testing of omadacycline against these pathogens.

## INTRODUCTION

Community-acquired bacterial pneumonia (CABP) is associated with considerable morbidity and mortality worldwide (1–4). Streptococcus pneumoniae and Haemophilus influenzae are leading causes of CABP (1, 4–7). Omadacycline, a novel tetracycline known as an aminomethylcycline, has been shown to have *in vitro* activity against Gram-positive and -negative organisms, including S. pneumoniae and H. influenzae (including resistant isolates) (8–12). Intravenous (i.v.) and oral (p.o.) formulations of omadacycline were approved in October 2018 by the U.S. Food and Drug Administration (FDA) for the treatment of patients with CABP and acute bacterial skin and skin structure infections (ABSSSI) (13).

Guidance for industry from the U.S. FDA (14) and the European Medicines Agency (EMA) (15) describe the benefit of evaluating pharmacokinetics (PK) and pharmacokinetic-pharmacodynamic (PK-PD) data during early- and late-stage development. During late-stage development of omadacycline, the collection of PK data in phase 3 clinical studies (16–19) proved useful to refine a previously-developed population PK model that was originally constructed using phase 1 data (20, 21). The Omadacycline for Pneumonia Treatment in the Community (OPTIC) study, a randomized, double-blind, active-comparator-controlled, multicenter study comparing i.v.-to-p.o. omadacycline and moxifloxacin in patients with CABP, was one such phase 3 study (17). Omadacycline met the U.S. FDA primary endpoint of noninferiority compared with moxifloxacin for early clinical response (ECR) assessed at 72 to 120 h following initiation of therapy in the intent-to-treat patient population. Omadacycline also met the EMA primary endpoint for noninferiority, which was investigator-assessed overall clinical response. This endpoint was assessed at the post-therapy evaluation (PTE), which occurred 5 to 10 days after the last dose of the study drug.

Use of nonclinical PK-PD data (22, 23), the above-described population PK model (21), and *in vitro* surveillance data (8) for omadacycline, together with Monte Carlo simulation, allowed for the conduct of PK-PD target attainment analyses. These analyses were conducted during late-stage development to provide support for i.v.-to-p.o. dosing regimens for the treatment of patients with CABP and interpretive criteria for the *in vitro* susceptibility testing of omadacycline against S. pneumoniae and H. influenzae. As described herein, these analyses were conducted using both total-drug epithelial lining fluid (ELF) and free-drug plasma exposures as well as four different approaches for selecting PK-PD targets, with the goal of comparing the results to patient outcomes by MIC.

## RESULTS

### Assessments for S. pneumoniae.

**FIG 1:**
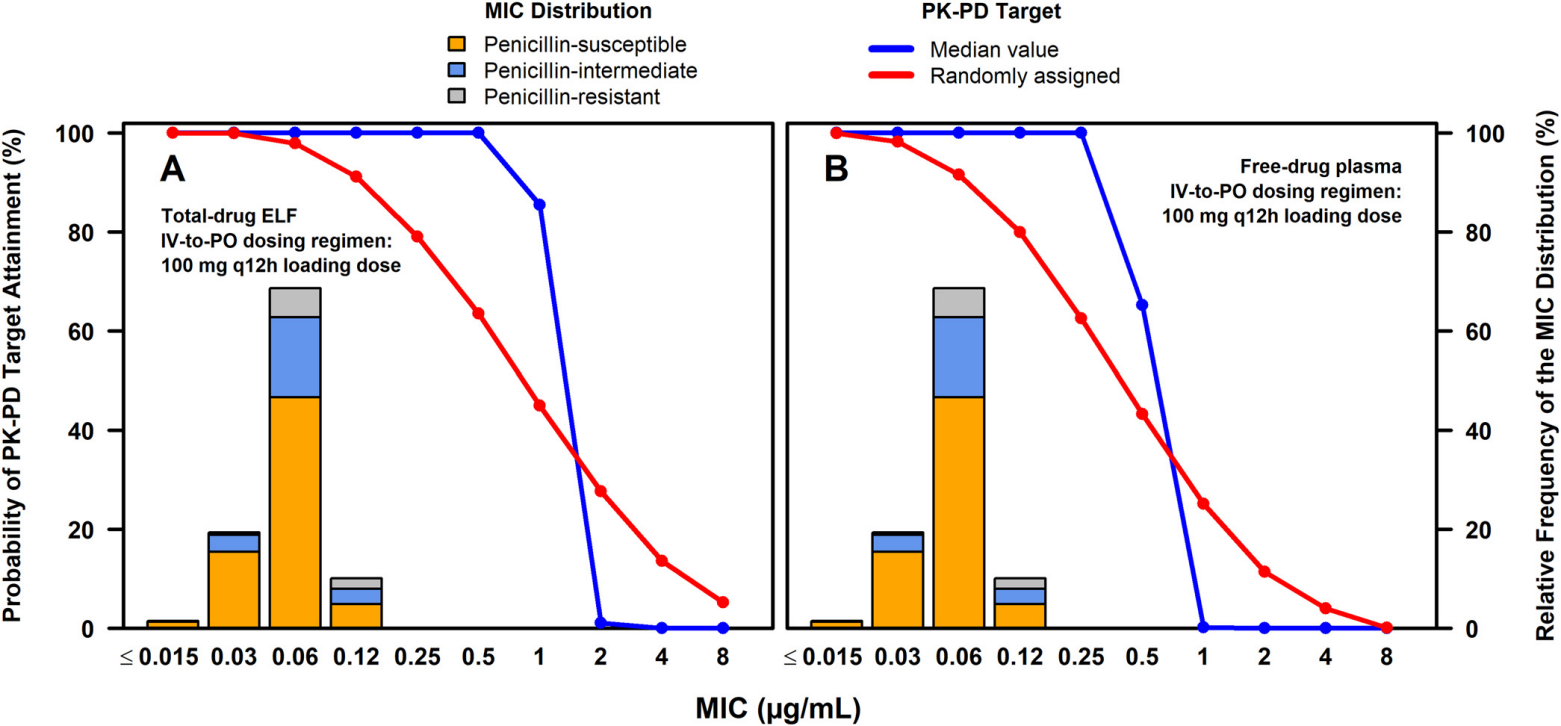
Percent probabilities of PK-PD target attainment by MIC on days 1 and 2 based on the evaluation of the total-drug ELF and free-drug plasma AUC/MIC ratio targets associated with a 1-log_10_ CFU reduction from baseline for S. pneumoniae (A and B, respectively) among simulated patients after the administration of omadacycline 100 mg i.v. q12h on day 1 followed by 100 mg i.v. q24h on day 2 and 300 mg p.o. q24h on days 3 to 5 overlaid on the MIC distribution for S. pneumoniae.

**(i) PK-PD target attainment assessments.** Percent probabilities of PK-PD target attainment by MIC on days 1 and 2 based on total-drug ELF or free-drug plasma ratio of the area of the concentration-time curve (AUC) to the MIC (AUC/MIC ratio) targets associated with a 1-log_10_ CFU reduction from baseline for S. pneumoniae from a neutropenic murine-lung infection model among simulated patients after the administration of omadacycline 100 mg i.v. every 12 h (q12h) on day 1 followed by 100 mg i.v. every 24 h (q24h) on day 2 and 300 mg p.o. q24h on days 3 to 5 are shown in Table 1. The observed percentage of successful ECR and clinical responses at PTE by MIC among omadacycline-treated patients with CABP and S. pneumoniae at baseline from the microbiological intent-to-treat (microITT) population of the phase 3 OPTIC study (17) are also shown in Table 1. Percent probabilities of PK-PD target attainment by MIC on days 1 and 2 based on randomly assigned and median total-drug ELF and free-drug plasma AUC/MIC ratio targets are shown overlaid on the MIC distribution for 1,314 S. pneumoniae isolates collected from medical centers in the United States and Europe in Fig. 1A and B for the above-described i.v.-to-p.o. dosing regimen, respectively. The stacked MIC bars in each panel show the proportions of penicillin-susceptible, -intermediate, and -resistant isolates.

**TABLE 1:** Comparison of observed percentages of successful clinical response by MIC among patients with CABP and S. pneumoniae at baseline and percent probabilities of PK-PD target attainment by MIC on days 1 and 2 based on total-drug ELF or free-drug plasma AUC/MIC ratio targets associated with a 1-log_10_ CFU reduction from baseline for S. pneumoniae among simulated patients after the administration of omadacycline 100 mg i.v. q12h on day 1 followed by 100 mg i.v. q24h on day 2 and 300 mg p.o. q24h on days 3 to 5

MIC (μg/mL)	% successful clinical response by MIC (no./total) for 28 patients^*a*^		% probability of PK-PD target attainment by MIC on days 1 and 2 among simulated patients^*b*^^,^^*c*^							
			Assessment of total-drug ELF exposures and AUC/MIC ratio targets				Assessment of free-drug plasma exposures and AUC/MIC ratio targets			
	ECR at 72 to 120 h	Clinical success at PTE	Randomly assigned based on all PK-PD targets^*d*^	Median of all PK-PD targets^*e*^	Second highest PK-PD target^*f*^	Highest PK-PD target^*g*^	Randomly assigned based on all PK-PD targets^*d*^	Median of all PK-PD targets^*e*^	Second highest PK-PD target^*f*^	Highest PK-PD target^*g*^
0.015	100 (2/2)	100 (2/2)	100	100	100	100	100	100	100	100
0.03	78.6 (11/14)	85.7 (12/14)	100	100	100	100	98.3	100	100	99.5
0.06	80.0 (8/10)	100 (10/10)	97.9	100	100	98.5	91.6	100	100	26.0
0.12	50.0 (1/2)	50 (1/2)	91.1	100	100	15.0	79.9	100	100	0
0.25	NA	NA	79.1	100	100	0	62.6	100	99.9	0
0.5	NA	NA	63.5	100	100	0	43.3	65.3	42.2	0
1	NA	NA	45.0	85.5	67.0	0	25.2	0.20	0.02	0
Overall^*h*^										
All	78.6 (22/28)	89.3 (25/28)	97.6	100	100	90.1	91.7	99.9	99.9	38.7
Pen-S			97.9	100	100	92.8	92.4	100	99.9	42.2
Pen-I			97.0	100	99.9	86.4	90.6	99.7	99.7	33.4
Pen-R			96.4	100	100	77.9	89.2	100	100	24.7

a Based on data from patients with CABP and S. pneumoniae at baseline in the microITT population of the phase 3 OPTIC study (17). NA, absence of data at a given MIC value.

b Assessed using total-drug ELF and free-drug plasma AUC/MIC ratio targets associated with a 1-log_10_ CFU reduction from baseline for S. pneumoniae based on data from a neutropenic murine-lung infection model (22).

c Based on the assessment of average 24-h total-drug ELF and free-drug plasma AUC values on days 1 and 2.

d Using data for all S. pneumoniae isolates studied, total-drug ELF and free-drug plasma AUC/MIC ratio targets associated with a 1-log_10_ CFU reduction from baseline were randomly assigned based on an estimated log normal distribution of AUC/MIC ratio targets associated with the same endpoint.

e Based on data for all S. pneumoniae isolates studied, the median total-drug ELF and free-drug plasma AUC/MIC ratio targets associated with a 1-log_10_ CFU reduction from baseline were 15.5 and 17.4, respectively (22).

f Based on data for all four S. pneumoniae isolates studied, the second highest total-drug ELF and free-drug plasma AUC/MIC ratio targets associated with a 1-log_10_ CFU reduction from baseline were 17.6 and 19.7, respectively (22).

g Based on data for all S. pneumoniae isolates studied, the highest total-drug ELF and free-drug plasma AUC/MIC ratio targets associated with a 1-log_10_ CFU reduction from baseline were 200.6 and 180.0, respectively (22).

h “Overall” represents the percentage of successful clinical response among all observed patients or the percent probability of PK-PD target attainment weighted over the given MIC distribution (8) for simulated patients. Pen-S, Pen-I, and Pen-R, penicillin susceptible, intermediate, and resistant, respectively.

At a MIC value of 0.12 μg/mL, the MIC_90_, high percent probabilities of PK-PD target attainment on days 1 and 2 based on total-drug ELF (91.1% for randomly assigned targets and 100% for both median and second highest targets) and free-drug plasma (79.9% for randomly assigned targets and 100% for both the median and second highest targets) AUC/MIC ratio targets were evident. At a MIC value of 0.25 μg/mL (i.e., the MIC value inhibiting 99.8% of S. pneumoniae isolates), percent probabilities of PK-PD target attainment on days 1 and 2 based on randomly assigned total-drug ELF and free-drug plasma AUC/MIC ratio targets were 79.1 and 62.6%, respectively. Percent probabilities of PK-PD target attainment on days 1 and 2 based on the median and second highest total-drug ELF and free-drug plasma AUC/MIC ratio targets ranged from 99.9 to 100%. As shown in Table S1 in the supplemental material, similar findings were evident for assessments carried out on day 3.

As shown in Table 1, overall percent probabilities of PK-PD target attainment on days 1 and 2 based on omadacycline MIC distributions for all S. pneumoniae isolates, and those for penicillin-susceptible, -intermediate, and -resistant subsets, ranged from 99.7% to 100% across the median and second highest total-drug ELF and free-drug plasma AUC/MIC ratio targets. For randomly assigned total-drug ELF and free-drug plasma AUC/MIC ratio targets, overall percent probabilities of PK-PD target attainment on days 1 and 2 ranged from 96.4 to 97.9% and 89.2 to 92.4%, respectively. For the highest total-drug ELF and free-drug plasma AUC/MIC ratio targets, overall percent probabilities ranged from 77.9 to 92.8% and 24.7 to 42.2%, respectively.

As shown in Table S1, overall percent probabilities of PK-PD target attainment on day 3 based on total-drug or free-drug plasma AUC/MIC ratio targets were similar to those on days 1 and 2, with the exception of reductions of approximately 15% relative to those on days 1 and 2 based on the highest total-drug ELF or free-drug plasma AUC/MIC ratio target.

Although presented, the percentages of all patients with S. pneumoniae at baseline with ECR at 72 to 120 h and clinical success at PTE, which were 78.6 and 89.3%, were not compared to the overall percent probabilities of PK-PD target attainment among simulated patients. While comparisons of percent probabilities of PK-PD target attainment and percentages of response by MIC value were carried out to assess correspondence by MIC value, the assessments of the percentages of ECR and clinical response at PTE and overall percent probabilities of PK-PD target attainment were not informative for this purpose.

As shown in Tables S2 and S3 and Fig. S1, the above-described evaluations of PK-PD target attainment for the omadacycline i.v.-to-p.o. dosing regimen containing the 100 mg i.v. q12h loading dose on day 1 were also carried out using total-drug ELF and free-drug plasma AUC/MIC ratio targets for S. pneumoniae, excluding the outlier S. pneumoniae isolate (S. pneumoniae 1293). As described in Materials and Methods, these assessments excluding this outlier isolate were undertaken given the differences in the distributions of total-drug ELF and free-drug plasma AUC/MIC ratio targets for S. pneumoniae with and without the outlier. At a MIC value of 0.25 μg/mL for S. pneumoniae, high percent probabilities of PK-PD target attainment on days 1 and 2 and on day 3 based on all total-drug ELF and free-drug plasma AUC/MIC ratio target assessments were noted (89.5% to 100%). Overall percent probabilities of PK-PD target attainment on days 1 and 2 and on day 3 based on all total-drug ELF and free-drug plasma AUC/MIC ratio target assessments, excluding the outlier, and MIC distributions for all S. pneumoniae isolates and penicillin-susceptible, -intermediate, and -resistant subsets, ranged from 99.6 to 100%.

As shown by the PK-PD target attainment results based on the highest total-drug ELF or free-drug plasma AUC/MIC ratio targets, there was a substantial improvement in the percent probabilities of PK-PD target attainment when considering the set of targets excluding the S. pneumoniae outlier. This was due to the large difference between the magnitude of the outlying AUC/MIC ratio target and the second highest target. The PK-PD target attainment results based on randomly assigned targets were also substantially improved by exclusion of the S. pneumoniae outlier due to the narrowing and general shift downward of the estimated log normal target distribution. Percent probabilities of PK-PD target attainment based on either the second highest or the median target were not substantially different between the two sets of analyses since there were only small differences in these fixed target values after exclusion of the outlier.

The above-described evaluations of PK-PD target attainment, based on total-drug ELF and free-drug plasma AUC/MIC ratio targets for S. pneumoniae determined including and excluding the outlier S. pneumoniae isolate, were also carried out to assess the omadacycline i.v.-to-p.o. dosing regimen containing the 200 mg i.v. q24h loading dose on day 1. Percent probabilities of PK-PD target attainment by MIC on days 1 and 2 and on day 3 among simulated patients after the administration of this dosing regimen are shown in Tables S4 and S5, respectively, based on AUC/MIC ratio targets including the outlier S. pneumoniae isolate, and Tables S6 and S7, respectively, based on AUC/MIC ratio targets excluding the outlier S. pneumoniae isolate. Figure S1 shows the percent probabilities of PK-PD target attainment by MIC on days 1 and 2 overlaid on the MIC distribution for S. pneumoniae for the omadacycline i.v.-to-p.o. dosing regimens containing the 100 mg i.v. q12h and 200 mg i.v. q24h loading doses on day 1. Percent probabilities of PK-PD target attainment at MIC values of 0.12 and 0.25 μg/mL and overall among simulated patients after the administration of omadacycline i.v.-to-p.o. dosing regimen containing the 200 mg i.v. q24h loading dose on day 1 were similar to those obtained after administration of the i.v.-to-p.o. dosing regimen containing the 100 mg i.v. q12h loading dose on day 1. These findings were expected given that the PK-PD index most closely associated with efficacy for tetracyclines is the AUC/MIC ratio (24–27) and that the day 1 total omadacycline dose was the same, yielding similar day 1 AUC values.

**FIG 2:**
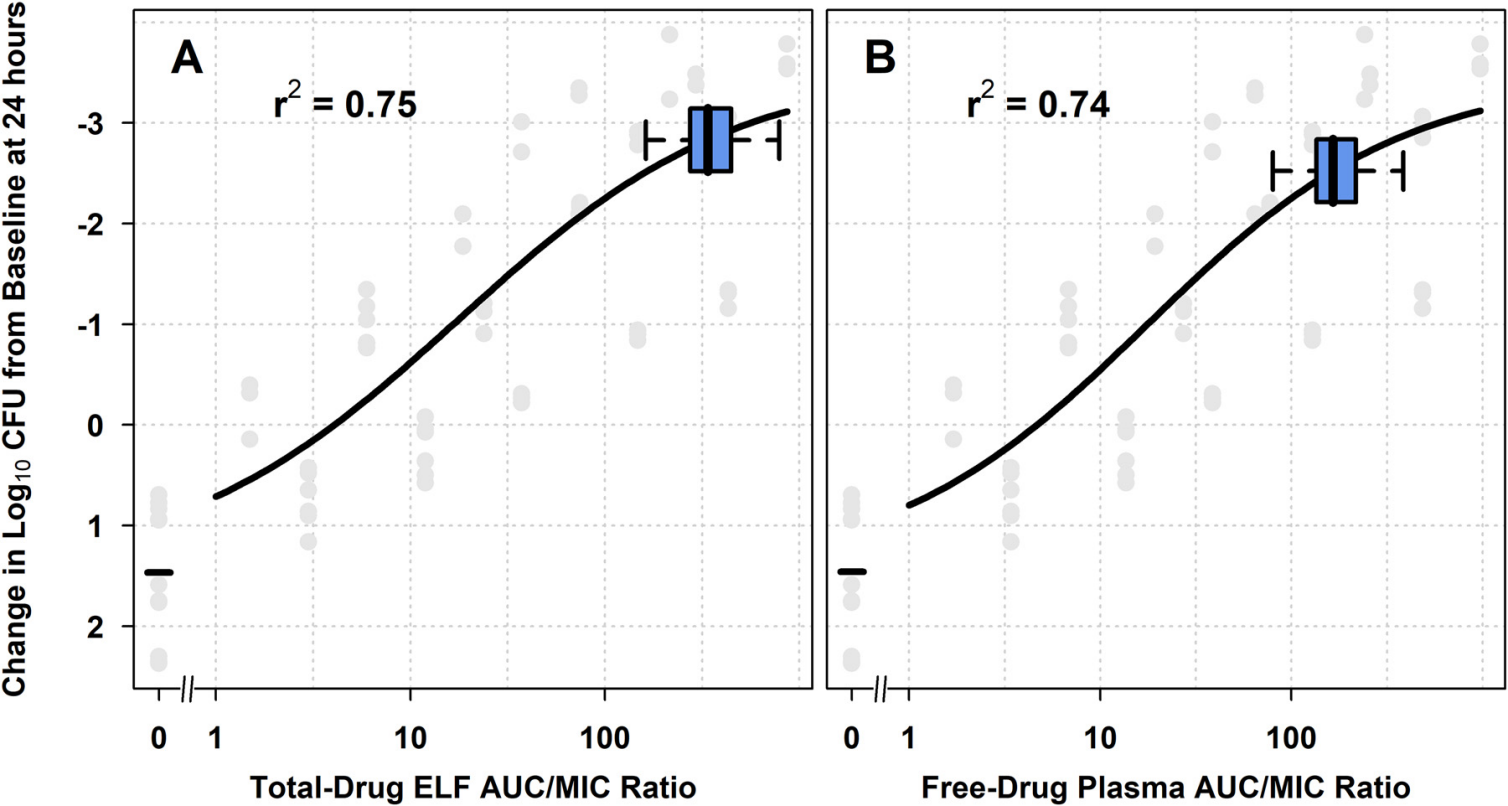
Nonclinical PK-PD relationships for efficacy for S. pneumoniae, overlaid with box-and-whisker plots of total-drug ELF and free-drug plasma AUC/MIC ratios on days 1 and 2 (A and B, respectively) for simulated patients after the administration of omadacycline 100 mg i.v. q12h on day 1 followed by 100 mg i.v. q24h on day 2 and 300 mg p.o. q24h on days 3 to 5. Horizontal box-and-whisker plots of total-drug ELF and free-drug plasma AUC/MIC ratios on days 1 and 2 for simulated patients after administration of omadacycline 100 mg i.v. q12h on day 1 followed by 100 mg i.v. q24h on day 2 and 300 mg p.o. q24h on days 3 to 5 are shown overlaid on the PK-PD relationship based on data from a neutropenic murine-lung infection model for S. pneumoniae. For each boxplot, the edge of the box represents the 25th to the 75th percentiles of the distribution for total-drug ELF or free-drug plasma AUC/MIC ratio. The line within the box represents the median total-drug ELF or free-drug plasma AUC/MIC ratio. The whiskers extend to the nearest value among those represented by 1.5× IQR of the box edges, where IQR is interquartile range as defined by the distribution of total-drug ELF or free-drug plasma AUC/MIC ratio from the 25th to the 75th percentiles.

**(ii) Assessments of nonclinical PK-PD relationships relative to AUC/MIC ratios among simulated patients.** Nonclinical PK-PD relationships for efficacy for S. pneumoniae relative to distributions for total-drug ELF and free-drug plasma AUC/MIC ratios among simulated patients were also assessed. Figure 2 shows the relationship between change in log_10_ CFU from baseline at 24 h and each of total-drug ELF and free-drug plasma AUC/MIC ratio on days 1 and 2, based on Hill-type models fit to data for all S. pneumoniae isolates studied using the neutropenic murine-lung infection model. Figure 2A and B show horizontal box-and-whisker plots of total-drug ELF and free-drug plasma AUC/MIC ratio, respectively, among simulated patients after the administration of omadacycline 100 mg i.v. q12h on day 1 followed by 100 mg i.v. q24h on day 2 and 300 mg p.o. q24h on days 3 to 5. The box-and-whisker plots demonstrate that the distributions of total-drug ELF and free-drug plasma AUC/MIC ratios among simulated patients were predominantly located on the upper portion of the estimated nonclinical PK-PD relationship, where a 2-log_10_ CFU or greater reduction from baseline was achieved.

The above-described nonclinical PK-PD relationships for S. pneumoniae with horizontal box-and-whisker plots of total-drug ELF and free-drug plasma AUC/MIC ratio among simulated patients on days 1 and 2 after the administration of the omadacycline i.v.-to-p.o. dosing regimen containing the 100 mg i.v. q12h loading dose on day 1 are shown in Fig. S2A and B, respectively. Similar such data are shown for the i.v.-to-p.o. dosing regimen containing the 200 mg i.v. q24h loading dose on day 1 in Fig. S2C and D, respectively. The box-and-whisker plots demonstrate that distributions of total-drug ELF and free-drug plasma AUC/MIC ratios on days 1 and 2 for simulated patients after administration of the omadacycline i.v.-to-p.o. dosing regimen containing the 200 mg i.v. q24h loading dose on day 1 were very similar to those for the i.v.-to-p.o. dosing regimen containing the 100 mg i.v. q12h loading dose on day 1.

### Assessments for H. influenzae.

**FIG 3:**
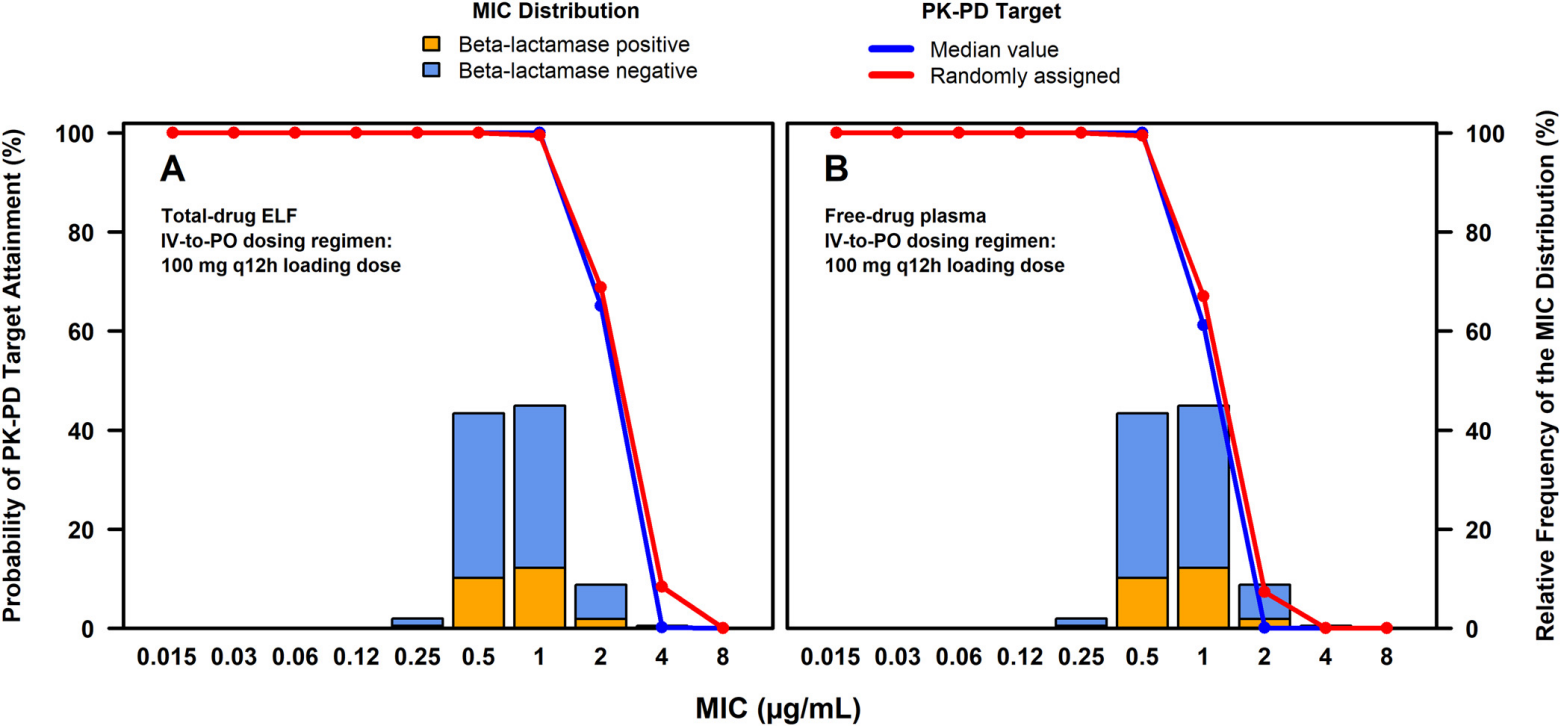
Percent probabilities of PK-PD target attainment by MIC on days 1 and 2 based on the evaluation of the total-drug ELF and free-drug plasma AUC/MIC ratio targets associated with a 1-log_10_ CFU reduction from baseline for H. influenzae (A and B, respectively) among simulated patients after the administration of omadacycline 100 mg i.v. q12h on day 1 followed by 100 mg i.v. q24h on day 2 and 300 mg p.o. q24h on days 3 to 5 overlaid on the MIC distribution for H. influenzae.

**(i) PK-PD target attainment assessments.** Percent probabilities of PK-PD target attainment by MIC on days 1 and 2 based on total-drug ELF or free-drug plasma AUC/MIC ratio targets associated with a 1-log_10_ CFU reduction from baseline for H. influenzae from a one-compartment *in vitro* infection model among simulated patients after the administration of omadacycline 100 mg i.v. q12h on day 1 followed by 100 mg i.v. q24h on day 2 and 300 mg p.o. q24h on days 3 to 5 are shown in Table 2. The observed percentage of successful ECR and clinical responses at PTE by MIC among omadacycline-treated patients with CABP and H. influenzae at baseline from the microITT population of the phase 3 OPTIC study are also shown in Table 2. Percent probabilities of PK-PD target attainment by MIC on days 1 and 2 based on randomly assigned and median total-drug ELF and free-drug plasma AUC/MIC ratio targets, overlaid on the MIC distribution for 803 H. influenzae isolates collected from medical centers in the United States and Europe, are shown in Fig. 3A and B, respectively. The stacked MIC bars in each panel show the proportions of β-lactamase-negative and -positive isolates.

**TABLE 2:** Comparison of observed percentages of successful clinical response by MIC among patients with CABP and H. influenzae at baseline and percent probabilities of PK-PD target attainment by MIC on days 1 and 2 based on total-drug ELF or free-drug plasma AUC/MIC ratio targets associated with a 1-log_10_ CFU reduction from baseline for H. influenzae among simulated patients after the administration of omadacycline 100 mg i.v. q12h on day 1 followed by 100 mg i.v. q24h on day 2 with a p.o. switch to 300 mg p.o. q24h on days 3 to 5

MIC (μg/mL)	% successful clinical response by MIC (no./total) for 32 patients^*a*^		% probability of PK-PD target attainment by MIC on days 1 and 2 among simulated patients^*b*^^,^^*c*^							
			Assessment of total-drug ELF exposures and AUC/MIC ratio targets				Assessment of free-drug plasma exposures and AUC/MIC ratio targets			
	ECR at 72 to 120 h	Clinical success at PTE	Randomly assigned based on all PK-PD targets^*d*^	Median of all PK-PD targets^*e*^	Second highest PK-PD target^*f*^	Highest PK-PD target^*g*^	Randomly assigned based on all PK-PD targets^*d*^	Median of all PK-PD targets^*e*^	Second highest PK-PD target^*f*^	Highest PK-PD target^*g*^
0.12	NA	NA	100	100	100	100	100	100	100	100
0.25	NA	NA	100	100	100	100	100	100	100	100
0.5	100 (1/1)	100 (1/1)	100	100	100	100	99.5	100	99.9	99.0
1	77.8 (14/18)	88.9 (16/18)	99.5	100	99.9	99.2	67.0	61.2	44.2	16.2
2	50.0 (6/12)	66.7 (8/12)	68.8	65.1	48.6	19.0	7.36	0.14	0.02	0
4	100 (1/1)	100 (1/1)	8.42	0.22	0.04	0	0	0	0	0
Overall^*h*^										
All	68.8 (22/32)	81.3 (26/32)	96.5	96.3	94.8	91.8	76.1	73.1	65.4	52.4
BL-Neg			96.4	96.2	94.7	91.6	76.2	73.2	65.8	53.2
BL-Pos			96.5	96.4	95.1	92.6	75.8	72.6	64.3	50.3

a Based on data from patients with CABP and H. influenzae at baseline in the microITT population of the phase 3 OPTIC study (17).

b Assessed using total-drug ELF/ free-drug plasma AUC/MIC ratio target associated with a 1-log_10_ CFU reduction from baseline for H. influenzae based on data from a one-compartment *in vitro* infection model (23).

c Based on the assessment of average 24-h total-drug ELF and free-drug plasma AUC values on Days 1 and 2.

d Using data for all H. influenzae isolates studied, total-drug ELF/free-drug plasma AUC/MIC ratio targets associated with a 1-log_10_ CFU reduction from baseline were randomly assigned based on an estimated log normal distribution of AUC/MIC ratio targets associated with the same endpoint.

e Based on data for all H. influenzae isolates studied, the median total-drug ELF/free-drug plasma AUC/MIC ratio target associated with a 1-log_10_ CFU reduction from baseline was 8.91 (23).

f Based on data for all H. influenzae isolates studied, the second highest total-drug ELF/free-drug plasma AUC/MIC ratio target associated with a 1-log_10_ CFU reduction from baseline was 9.73 (23).

g Based on data for all H. influenzae isolates studied, the highest total-drug ELF/free-drug plasma AUC/MIC ratio target associated with a 1-log_10_ CFU reduction from baseline was 11.6 (23).

h “Overall” represents the percentage of successful clinical response among all observed patients or the percent probability of PK-PD target attainment weighted over the given MIC distribution (8) for simulated patients. BL-Neg and BL-Pos, beta-lactamase negative and positive, respectively.

At a MIC value of 0.5 μg/mL for H. influenzae (i.e., the MIC value inhibiting 45.6% of isolates), percent probabilities of PK-PD target attainment on days 1 and 2 based on total-drug ELF AUC/MIC ratio targets for all four approaches were 100%. For the assessment of free-drug plasma AUC/MIC ratio targets, high percent probabilities of PK-PD target attainment (99.5 to 100%) on days 1 and 2 were evident for all four approaches. At a MIC_90_ value of 1 μg/mL for H. influenzae, percent probabilities of PK-PD target attainment based on total-drug ELF AUC/MIC ratio targets ranged from 99.2 to 100% for all four approaches; for the evaluation of free-drug plasma AUC/MIC ratio targets, percent probabilities of PK-PD target attainment ranged from 44.2 to 67.0% for all approaches except for the highest target, the percent probability for which was 16.2%.

As shown in Table 2, overall percent probabilities of PK-PD target attainment on days 1 and 2 based on omadacycline MIC distributions for all H. influenzae isolates, and those for beta-lactamase-negative and -positive subsets, ranged from 91.6 to 96.5% across all total-drug ELF AUC/MIC ratio targets. Across all free-drug plasma AUC/MIC ratio targets, overall percent probabilities of PK-PD target attainment ranged from 50.3 to 76.2%.

As shown in Table S8, percent probabilities of PK-PD target attainment at a MIC value of 0.5 μg/mL on day 3 based on total-drug ELF AUC/MIC ratio targets for H. influenzae were similar to those for days 1 and 2. However, percent probabilities of PK-PD target attainment based on free-drug plasma AUC/MIC ratio targets at the same MIC value were 5.9 to 22.6% lower on day 3 than on days 1 and 2. Overall percent probabilities of PK-PD target attainment on day 3 based on omadacycline MIC distributions for all H. influenzae isolates, and those for beta-lactamase-negative and -positive subsets, were 4.8 to 19.3% lower than those for days 1 and 2 across all total-drug ELF and free-drug plasma AUC/MIC ratio targets.

As described for S. pneumoniae, the percentages of all patients with H. influenzae at baseline with ECR at 72 to 120 h and clinical success at PTE, which were 68.8 and 80.6%, were not compared to the overall percent probabilities of PK-PD target attainment among simulated patients. While comparisons of percent probabilities of PK-PD target attainment and percentages of response by MIC were carried out to assess correspondence by MIC, the assessments of the percentages of ECR and clinical response at PTE and overall percent probabilities of PK-PD target attainment were not informative for this purpose.

The above-described evaluations of PK-PD target attainment were also carried out to assess the omadacycline i.v.-to-p.o. dosing regimen containing the 200 mg i.v. q24h loading dose on day 1. Percent probabilities of PK-PD target attainment by MIC on days 1 and 2 and on day 3 among simulated patients after the administration of this dosing regimen are shown in Tables S9 and S10, respectively, and those on days 1 and 2 overlaid on the MIC distribution for H. influenzae are shown in Fig. S3. Percent probabilities of PK-PD target attainment at MIC values of 1 and 0.5 μg/mL and overall among simulated patients after the administration of omadacycline 200 mg i.v. q24h on day 1 followed by 100 mg i.v. q24h on day 2 and 300 mg p.o. q24h on days 3 to 5 were similar to those obtained after administration of the i.v.-to-p.o. dosing regimen with 100 mg i.v. q12h on day 1. As described above for S. pneumoniae, these results were expected given the PK-PD characteristics for tetracyclines and same total day 1 total omadacycline dose.

**FIG 4:**
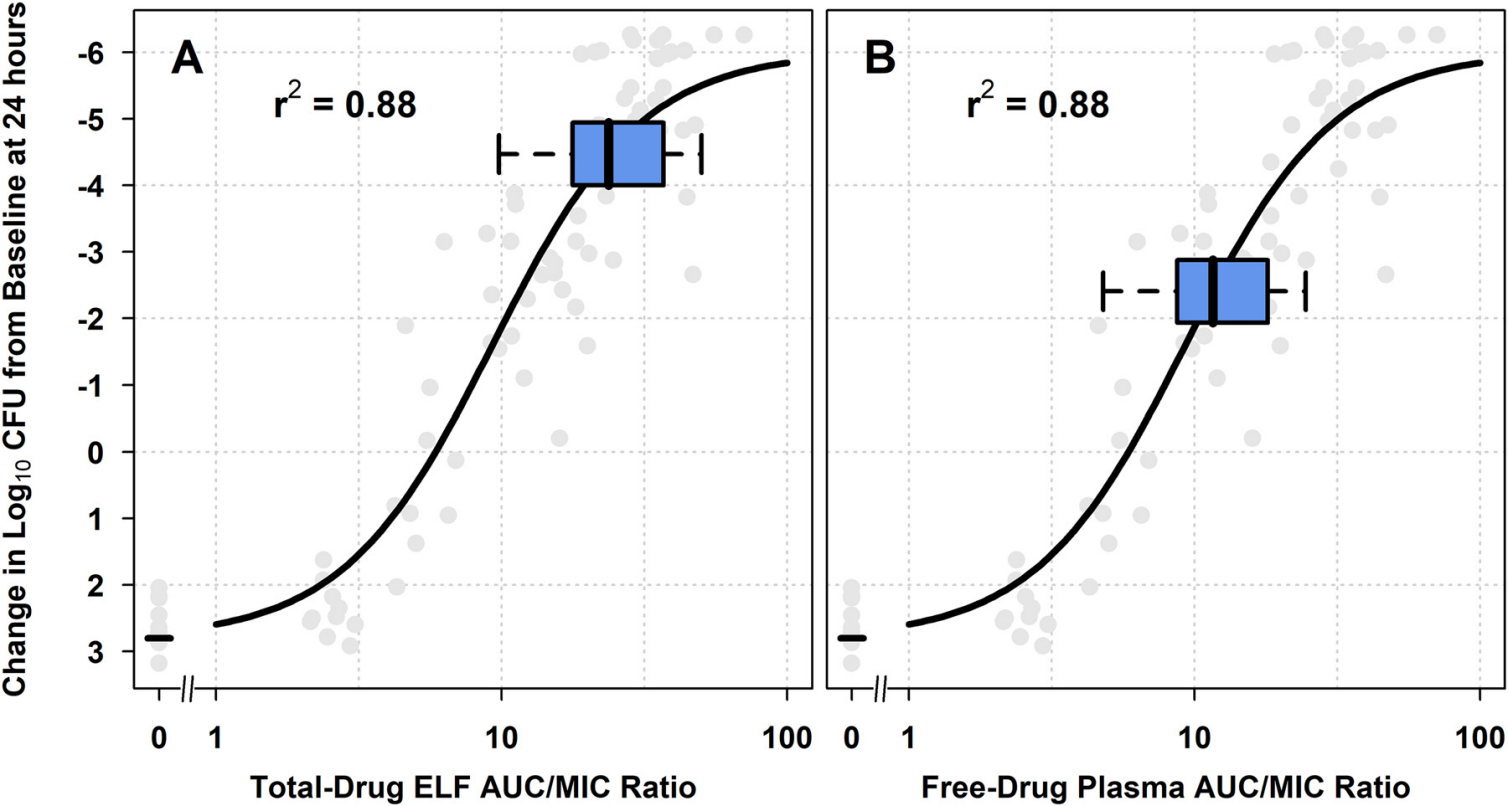
Nonclinical PK-PD relationships for efficacy for H. influenzae, overlaid with box-and-whisker plots of total-drug ELF and free-drug plasma AUC/MIC ratios on days 1 and 2 (A and B, respectively) for simulated patients after administration of omadacycline 100 mg i.v. q12h on day 1 followed by 100 mg i.v. q24h on day 2 and 300 mg p.o. q24h on days 3 to 5. Horizontal box-and-whisker plots of total-drug ELF and free-drug plasma AUC/MIC ratios on days 1 and 2 for simulated patients after administration of omadacycline 100 mg i.v. q12h on day 1 followed by 100 mg i.v. q24h on day 2 and 300 mg p.o. q24h on days 3 to 5 are shown overlaid on the PK-PD relationship based on data from a one-compartment *in vitro* infection model for H. influenzae. For each boxplot, the edge of the box represents the 25th to the 75th percentiles of the distribution for total-drug ELF or free-drug plasma AUC/MIC ratio. The line within the box represents the median total-drug ELF or free-drug plasma AUC/MIC ratio. The whiskers extend to the nearest value among those represented by 1.5× IQR of the box edges.

**(ii) Assessments of nonclinical PK-PD relationships relative to AUC/MIC ratios among simulated patients.** Figure 4 shows the relationships between change in log_10_ CFU from baseline at 24 h and each of total-drug ELF and free-drug plasma AUC/MIC ratio, based on Hill-type models fit to data for H. influenzae isolates studied using the one-compartment *in vitro* infection model. Figure 4A and B show the horizontal box-and-whisker plots of total-drug ELF and free-drug plasma AUC/MIC ratio, respectively, among simulated patients after the administration of omadacycline 100 mg i.v. q12h on day 1 followed by 100 mg i.v. q24h on day 2 and 300 mg p.o. q24h on day 3. The box-and-whisker plots demonstrated that the distribution of total-drug ELF AUC/MIC ratios among simulated patients was almost completely located on the upper portion of the estimated nonclinical PK-PD relationship, where a 2-log_10_ CFU or greater reduction from baseline was achieved, while barely a majority of the distribution for free-drug plasma AUC/MIC ratios were in the region where 2-log_10_ CFU or greater reduction from baseline was achieved. Figure S4 shows the above-described nonclinical PK-PD relationships for H. influenzae with horizontal box-and-whisker plots of total-drug ELF and free-drug plasma AUC/MIC ratio on days 1 and 2 for simulated patients after the administration of omadacycline 100 mg i.v. q12h on day 1 followed by 100 mg i.v. q24h on day 2 and 300 mg p.o. q24h on days 3 to 5 in panels A and B, respectively, and 200 mg i.v. q24h on day 1 followed by 100 mg i.v. q24h on day 2 and 300 mg p.o. q24h on days 3 to 5 in panels C and D, respectively. The box-and-whisker plots demonstrate that distributions of total-drug ELF and free-drug plasma AUC/MIC ratios on days 1 and 2 for simulated patients after administration of the omadacycline i.v.-to-p.o. dosing regimen containing the 200 mg i.v. q24h loading dose on day 1 were very similar to those for the i.v.-to-p.o. dosing regimen containing the 100 mg i.v. q12h loading dose on day 1.

## DISCUSSION

The objective of these analyses was to evaluate the agreement by MIC between percent probabilities of PK-PD target attainment among simulated omadacycline-treated patients and observed clinical response among omadacycline-treated patients with CABP. PK-PD target attainment analyses, which were undertaken to provide support for omadacycline i.v.-to-p.o. dosing regimens, were carried out using two drug exposure measures and four sets of PK-PD targets for the same nonclinical endpoint. Drug exposure measures evaluated were total-drug ELF and free-drug plasma AUC values. For dose selection, it is important to consider effect site exposure (28–30), and consequently, the results using the total-drug ELF AUC values were emphasized herein. PK-PD targets for efficacy evaluated included the median, second highest, and highest total-drug ELF and free-drug plasma AUC/MIC ratio targets associated with a 1-log_10_ CFU reduction from baseline for S. pneumoniae and H. influenzae. Additionally, randomly assigned total-drug ELF and free-drug plasma AUC/MIC ratio targets for the above-described endpoint, an approach which accounts for variability in PK-PD targets, were evaluated.

A 1-log_10_ CFU reduction from baseline was chosen for evaluation given the results of prior PK-PD target attainment analyses conducted to evaluate antimicrobial dosing regimens for the treatment of patients with pneumonia. Results of this assessment demonstrated a relationship between achievement PK-PD targets associated with a 1-log_10_ CFU reduction from baseline and an increased probability of a successful regulatory outcome (i.e., pivotal clinical trials which demonstrated noninferiority) (31). Additionally, others have recommended a 1-log_10_ CFU reduction from baseline as an endpoint for evaluation for infections associated with higher bacterial inocula, such as pneumonia (15, 29).

The results of PK-PD target attainment analyses on days 1 and 2 at the MIC_50_ and MIC_90_ values for S. pneumoniae (0.06 and 0.12 μg/mL, respectively) and H. influenzae (MIC_50/90_ value = 1 μg/mL) among simulated patients after administration of the omadacycline i.v.-to-p.o. dosing regimen with 100 mg i.v. q12h on day 1 were compared to the percentage of patients with ECR at 72 to 120 h and clinical success at PTE at the same MIC values. For S. pneumoniae, percent probabilities of PK-PD target attainment on days 1 and 2 based on all total-drug ELF and free-drug plasma AUC/MIC ratio targets, with the exception of the highest free-drug plasma target, were generally concordant with percentages of successful response for the two clinical endpoints. At the MIC_50_ value of 0.06 μg/mL, the percent probability of PK-PD target attainment based on the highest free-drug plasma AUC/MIC ratio target was 26.0%; for all other AUC/MIC ratio targets, percent probabilities ranged from 97.9 to 100%. The percentages of the 10 patients with ECR at 72 to 120 h and clinical success at PTE at the MIC_50_ value were 80.0 and 100%, respectively. The low percent probability of PK-PD target attainment for the highest free-drug plasma AUC/MIC ratio target was not surprising given that this target was approximately 13-fold higher than that of the median, a finding attributed to the assumption that S. pneumoniae 1293 was an outlier. Given that only two patients with CABP had a baseline S. pneumoniae isolate with a MIC_90_ value of 0.12 μg/mL, comparisons between simulated and clinical data were not possible.

For H. influenzae, percent probabilities of PK-PD target attainment based on total-drug ELF AUC/MIC ratio targets were generally more concordant with percentages of successful response for the two clinical endpoints than those based on free-drug plasma AUC/MIC ratio targets. For H. influenzae, percent probabilities of PK-PD target attainment at a MIC value of 1 μg/mL based on total-drug ELF AUC/MIC ratio targets ranged from 99.2 to 100%, in contrast to 16.2 to 67.0% based on free-drug plasma AUC/MIC ratio targets. The percentages of the 18 patients with ECR at 72 to 120 h and clinical success at PTE at the same MIC value was 77.8 and 88.9%, respectively. At a MIC value of 2 μg/mL, for which the percentages of 12 patients with ECR at 72 to 120 h and clinical success at PTE were 50.0 and 66.7%, respectively, differences in percent probabilities of PK-PD target attainment among the four approaches using total-drug ELF AUC/MIC ratio targets were more apparent. For randomly assigned and median targets, percent probabilities of PK-PD target attainment were 68.8 and 65.1%, respectively; for the second highest and highest total-drug ELF AUC/MIC ratio targets, percent probabilities of PK-PD target attainment were 48.6 and 19.0%, respectively. Percent probabilities of PK-PD target attainment based on all four approaches using free-drug plasma AUC/MIC ratio targets were ≤7.36%. This basis for the above-described closer concordance between clinical data and results of PK-PD target attainment analyses for H. influenzae based on randomly assigned and median total-drug ELF rather than free-drug plasma AUC/MIC ratio targets is likely a function of the ELF penetration ratio of 2.06 (21), an indicator that omadacycline exposure is higher at the effect site than in plasma.

It is important to recognize the limitations of the above-described comparisons between simulated and clinical data. The assessments of clinical response by MIC were univariable and thus, did not consider other factors, such as underlying comorbidities, that may contribute to patient response. In addition, the comparisons, which were based on a limited number of patients, may be more susceptible to type II errors. These limitations likely obscured the ability to further discriminate between the results of PK-PD target attainment analyses and assessments of clinical response by MIC. Regardless, these data provide support for the premise that results of PK-PD target attainment based on the highest PK-PD target are not sufficiently predictive of clinical outcome, even if the PK-PD targets for a collection of isolates do not contain an extreme outlier, as was the case with the assessment for H. influenzae. The above-described analyses of antimicrobial dosing regimens for the treatment of patients with pneumonia provide support for the evaluation of a PK-PD target for efficacy that is representative of the central tendency for the distribution of PK-PD targets (31). However, the limitation of using a fixed PK-PD target, even the median value, is the lack of consideration of interisolate variability. Random assignment of PK-PD targets, which represents the least biased approach for conducting PK-PD target attainment analyses to support dose selection, is analogous to the existing practice of assigning drug exposure. The use of Monte Carlo simulation in this case accounts for interindividual variability in drug exposure given that each simulated patient is randomly assigned PK parameter estimates (e.g., clearance and volume of distribution terms) based on frequency distributions for those PK parameters.

When using a fixed median PK-PD target, the PK-PD target attainment results may be overestimated, while the use of the maximum PK-PD target or other high percentiles, such as the second highest, for the PK-PD target distribution may lead to underestimated results. The risk of the latter is the selection of dosing regimens with higher total daily doses and, thus, poor safety profiles. The benefit of randomly assigning a PK-PD target is that high PK-PD targets are considered but according to a probability distribution of the expected occurrence of such targets. It should be noted that there are no current standard recommendations describing which approach for PK-PD targets for efficacy to assess when conducting PK-PD target attainment analyses. However, as described above, the use of randomly assigned PK-PD targets addresses the concern of appropriately considering higher PK-PD targets. Thus, we suggest that this approach be considered the standard for selecting PK-PD targets for efficacy when assessing the adequacy of dosing regimens and interpretive criteria for *in vitro* susceptibility testing.

The assessment of nonclinical PK-PD data using Hill models relative to the distribution of PK-PD indices in simulated patients is another useful approach to evaluate the adequacy of selected dosing regimens. As shown in Fig. 2 and 4, the majority of simulated patients achieved total-drug ELF and free-drug plasma AUC/MIC ratios associated with a 1-log_10_ CFU or greater reduction from baseline. This magnitude of bacterial reduction was consistent with that for other antimicrobial agents that are used successfully to treat patients with pneumonia (32, 33). Thus, the evaluation of nonclinical data relative to simulated PK-PD data in this manner also provided support for the omadacycline i.v.-to-p.o. dosing regimen selected for patients with CABP.

In addition to dose support, results of PK-PD target attainment analyses and assessments of clinical response by MIC were useful to support interpretive criteria decisions for *in vitro* susceptibility testing of omadacycline against S. pneumoniae and Haemophilus species. The results of both assessments provided support for the U.S. FDA interpretive criteria for S. pneumoniae of ≤0.12, 0.25, and ≥0.5 μg/mL for susceptible, intermediate, and resistant, respectively (34). The U.S. FDA interpretive criteria for *in vitro* susceptibility testing of omadacycline against Haemophilus species, which are ≤2, 4, and ≥8 μg/mL for susceptible, intermediate, and resistant, respectively, were however, supported by the epidemiological cutoff value, which was 2 μg/mL (https://paratek-keystone.com/), rather than the results of the PK-PD target attainment analyses.

In conclusion, results of the various assessments described herein served to provide support for omadacycline dosing regimens for the treatment of patients with CABP and decisions about the interpretive criteria for *in vitro* susceptibility testing of omadacycline against S. pneumoniae and H. influenzae. Such data also served to demonstrate the benefit accounting for variability in PK-PD targets through random assignment rather than assessing fixed PK-PD targets.

## MATERIALS AND METHODS

### Population pharmacokinetic model.

A previously-developed population PK model based on intravenous and oral PK data from phase 1 studies and infected patients was used (21). Data used to develop the population PK model were from 13 phase 1 studies, a phase 1b uncomplicated urinary tract infection study (35), one phase 3 CABP study (17), and two phase 3 ABSSSI studies (16, 18). This model was subsequently assessed using an external third phase 3 study conducted in patients with ABSSSI (19). The final population PK model was a three-compartment model with first-order absorption using transit compartments to account for absorption delay following oral dosing and first-order elimination. ELF concentrations, based on data from one of the phase 1 studies evaluated (36), were modeled as a subcompartment of the first peripheral compartment. Based on the generation of day 4 AUC from time zero to 24 h using Monte Carlo simulation, the ratio of the total-drug ELF AUC to free-drug plasma AUC was determined to be 2.06.

### Monte Carlo simulations.

Using the above-described population PK model (21) and the covariate, sex, which was assigned to simulated patients in equal proportions, individual *post hoc* parameter estimates were generated for 5,000 simulated patients using the mrgsolve package in R version 3.3.1 (37, 38). Using these *post hoc* parameter estimates, total-drug plasma and ELF concentration-time profiles from 0 to 120 h were generated for each simulated patient after administration of omadacycline 100 mg i.v. q12h on day 1 followed by 100 mg i.v. q24h on day 2 and 300 mg p.o. q24h on days 3 to 5 (as evaluated in the phase 3 OPTIC study [17]). In addition, the omadacycline i.v.-to-p.o. dosing regimen approved by the U.S. FDA (13) that provides the same exposure, 200 mg i.v. q24h on day 1 followed by 100 mg i.v. on day 2 and 300 mg p.o. q24h on days 3 to 5, was also evaluated.

Average 24-h total-drug plasma and ELF AUC values on days 1 and 2 were calculated by numerical integration of the total-drug plasma concentration curves from 0 to 48 h and then division of the resulting AUC by 2. Additionally, 24-h total-drug plasma AUC values were determined after the p.o. switch on day 3 for i.v.-to-p.o. dosing regimens. Total-drug plasma AUC values were adjusted to free-drug plasma AUC values using a free fraction of 0.79 based on *in vitro* data for human plasma protein binding (39).

Free-drug plasma and total-drug ELF AUC values for each simulated patient were divided by MIC values, the range for which was determined based on the observed *in vitro* surveillance data for omadacycline against S. pneumoniae and H. influenzae (8). As described previously, AUC/MIC ratio is generally considered the PK-PD index most closely associated with efficacy for tetracyclines (24–27).

### Nonclinical pharmacokinetic-pharmacodynamic targets for efficacy.

Nonclinical total-drug ELF AUC/MIC ratio targets for omadacycline against S. pneumoniae and H. influenzae based on data from neutropenic murine-lung and one-compartment *in vitro* infection models, respectively, were evaluated (22, 23). In Table S11, the median free-drug plasma and total-drug ELF AUC/MIC ratio targets associated with a 1-log_10_ CFU reduction from baseline for S. pneumoniae are presented based on data including and excluding isolate 1293, the data for which were considered to be an outlier. While the median values were similar (total-drug ELF AUC/MIC ratio of 15.5 versus 13.3; free-drug plasma AUC/MIC ratio of 17.4 versus 15.2), the mean value including isolate 1293 was over four times greater than the mean value excluding isolate 1293 (total-drug ELF AUC/MIC ratio of 59.4 versus 12.3; free-drug plasma AUC/MIC ratio of 55.2 versus 13.6). Common reasons for the occurrence of such an outlier can be poor reproducibility of MIC values (i.e., measurement error) at the MIC value for this isolate (MIC = 0.06 μg/mL), resistance emergence on therapy (which is difficult to detect in a short-duration *in vivo* experiment), and differences in the way the bacterial inoculum was delivered to the animal (experimental error). The investigators of the murine-lung study noted the relatively large value of the free-drug plasma AUC/MIC ratio associated with a 1-log_10_ CFU reduction from baseline for S. pneumoniae 1293 but could not explain the basis for the outlier given the above potential reasons (D. Andes, personal communication). Thus, PK-PD target attainment analyses were carried out with and without consideration of the data for S. pneumoniae 1293. While both sets of results were considered, emphasis was placed on the results including the outlier. As shown in Table S12, the median total-drug ELF/free-drug plasma AUC/MIC ratio target associated with a 1-log_10_ CFU reduction from baseline for H. influenzae was 8.91.

Four approaches for selecting total-drug ELF and free-drug plasma AUC/MIC ratio targets associated with a 1-log_10_ CFU reduction from baseline for the PK-PD target attainment analyses were employed. These included randomly assigned and the median, second highest, and highest total-drug ELF or free-drug plasma AUC/MIC ratio targets. Randomly assigned AUC/MIC ratio targets were based on an estimated log normal distribution derived from the targets associated with a 1-log_10_ CFU reduction from baseline described in Tables S11 and S12. The distribution was truncated at ±2 standard deviations on the log scale. Using S. pneumoniae as an example, Fig. S5 shows the estimated truncated log normal distributions of free-drug plasma AUC/MIC ratio targets associated with a 1-log_10_ CFU reduction from baseline for S. pneumoniae from a neutropenic murine-lung infection model, with and without consideration of the outlier (S. pneumoniae 1293).

### Pharmacokinetic-pharmacodynamic target attainment analyses.

PK-PD target attainment analyses were performed using R version 3.3.1 (38). Percent probabilities of PK-PD target attainment by MIC were assessed based on the above-described four approaches for selection of total-drug ELF or free-drug plasma AUC/MIC ratio targets. Percent probabilities of PK-PD target attainment by MIC were assessed on days 1 and 2 using average 24-h total-drug ELF and free-drug plasma AUC values on days 1 and 2 and the 24-h total-drug ELF and free-drug plasma AUC values on day 3 (i.e., the earliest day of p.o. switch for i.v.-to-p.o. dosing regimens) after administration of each omadacycline dosing regimen among simulated patients.

Percent probabilities of PK-PD target attainment by MIC on the above-described days of assessment were compared to the observed percentage of successful clinical responses by MIC among omadacycline-treated patients with CABP and S. pneumoniae or H. influenzae at baseline in the microITT population of the phase 3 study (17). In this study, omadacycline-treated patients received two doses of omadacycline 100 mg i.v. q12h followed by 100 mg i.v. q24h, with the option to switch to 300 mg p.o. q24h after at least 3 days of i.v. treatment. The duration of treatment was 7 to 14 days. Efficacy endpoints assessed included ECR, which was evaluated at 72 to 120 h after administration of the first dose of study drug, and clinical success at the PTE visit, which occurred 5 to 10 days after the last dose of the study drug.

Percent probabilities of PK-PD target attainment were also assessed weighted over omadacycline MIC distributions for 1,314 S. pneumoniae and 803 H. influenzae isolates collected during 2016 from medical centers in the United States and Europe that were part of the SENTRY Antimicrobial Surveillance Program (8). The MIC_50_ and MIC_90_ values were 0.06 and 0.12 μg/mL, respectively, for S. pneumoniae; for H. influenzae, both values were 1 μg/mL. Percent probabilities of PK-PD target attainment weighted over omadacycline MIC distributions were determined by multiplying the percent probability of PK-PD target attainment for a given AUC/MIC ratio target at a given MIC value by the probability of occurrence of that MIC value and then taking the sum of these products over all MIC values. Overall percent probabilities of PK-PD target attainment were also assessed for the subset of S. pneumoniae isolates that were penicillin susceptible, intermediate, and resistant (*n* = 899, 263, and 152, respectively) and H. influenzae isolates that were beta-lactamase negative and positive (*n* = 602 and 201, respectively).

Emphasis was placed on the PK-PD target attainment results based on total-drug ELF AUC/MIC ratio targets associated with a 1-log_10_ CFU reduction from baseline. The choice to focus examination on the results for this endpoint was supported by results of previous PK-PD target attainment analysis for antibacterial dosing regimens that were evaluated for pneumonia. Results of this analysis demonstrated a relationship between the percent probability of achieving a PK-PD target associated with a 1-log_10_ CFU reduction from baseline and probability of a successful regulatory outcome, the latter of which was an indicator of meeting noninferiority in pivotal clinical trials. An increased percent probability of PK-PD target attainment was associated with an increased probability of a successful regulatory outcome. Additionally, emphasis was placed on results for total-drug ELF instead of free-drug plasma exposures given the importance of considering effect site exposures (28–30).

### Evaluation of nonclinical PK-PD relationships for efficacy relative to AUC/MIC ratios among simulated patients.

Using parameter estimates from Hill models constructed using raw data for all the S. pneumoniae and H. influenzae isolates (22, 23) summarized in Tables S11 and S12, respectively, fitted functions for the relationship between change in log_10_ CFU from baseline at 24 h and total-drug ELF or free-drug plasma AUC/MIC ratio were generated for each pathogen and omadacycline dosing regimen evaluated. Total-drug ELF AUC/MIC ratios for simulated patients were generated by taking the average total-drug ELF and free-drug plasma AUC values for days 1 and 2 for each simulated patient after administration of omadacycline i.v.-to-p.o. dosing regimens and dividing these AUC values by a MIC value that was randomly assigned from the above-described MIC distributions for isolates for S. pneumoniae and H. influenzae (8). To enable interpretation of the nonclinical PK-PD relationships for efficacy relative to total-drug ELF AUC/MIC ratios for simulated patients, box-and-whisker plots of total-drug ELF AUC/MIC ratios were overlaid on the above-described fitted functions. These assessments were carried out using R version 3.3.1 (38).
