# Supplementary material for: Pharmacokinetic-Pharmacodynamic Target Attainment Analyses Evaluating Omadacycline Dosing Regimens for the Treatment of Patients with Community-Acquired Bacterial Pneumonia Arising from Streptococcus pneumoniae and Haemophilus influenzae
Source: Antimicrob Agents Chemother. 2023 Mar 22;67(4):e02213-21. doi: 10.1128/aac.02213-21 (PMC10112269; doi:10.1128/aac.02213-21)
Supplement: Supplemental file 1 — Supplemental material. Download aac.02213-21-s0001.pdf, PDF file, 1.7 MB [file aac.02213-21-s0001.pdf]

Table S1. Comparison of observed percentages of successful clinical response by MIC among patients with CABP and *S. pneumoniae* at baseline and percent probabilities of PK-PD target attainment by MIC on day 3 based on total-drug ELF and free-drug plasma AUC/MIC ratio targets associated with a 1-$\log_{10}$ CFU reduction from baseline for *S. pneumoniae,* including the outlier isolate, among simulated patients after the administration of omadacycline 100 mg i.v. q12h on day 1 followed by 100 mg i.v. q24h on day 2 and 300 mg p.o. q24h on days 3 to 5

MIC (µg/mL)	% of successful clinical response by MIC (no./total) for 28 patients[a]		Percent probability of PK-PD target attainment by MIC on day 3 among simulated patients[b,c]							
			Assessment of total-drug ELF exposures and AUC/MIC ratio targets				Assessment of free-drug plasma exposures and AUC/MIC ratio targets			
	ECR at 72 to 120 hours	Clinical success at PTE	Randomly assigned based on PK-PD targets[d]	Median of PK-PD targets[e]	Second highest PK-PD target[f]	Highest PK-PD target[g]	Randomly assigned based on PK-PD targets[d]	Median of PK-PD targets[e]	Second highest PK-PD target[f]	Highest PK-PD target[g]
0.015	100 (2/2)	100 (2/2)	100	100	100	100	99.6	100	100	99.9
0.03	78.6 (11/14)	85.7 (12/14)	99.5	100	100	99.8	96.0	100	100	82.8
0.06	80.0 (8/10)	100 (10/10)	95.7	100	100	76.7	87.6	100	100	10.4
0.12	50.0 (1/2)	50 (1/2)	87.5	100	100	6.52	74.1	100	100	0.06
0.25	NA	NA	73.8	100	100	0	55.1	94.9	89.5	0
0.5	NA	NA	56.8	98.3	95.9	0	36.4	28.8	17.3	0
1	NA	NA	38.5	47.1	31.2	0	20.0	0.24	0.12	0
**Overall[h]**										
All	78.6 (22/28)	89.3 (25/28)	95.6	100	99.9	74.2	87.9	99.9	99.8	24.7
Pen-S			96.0	100	100	77.3	88.7	99.9	99.9	27.8
Pen-I			94.9	99.8	99.8	69.9	86.6	99.6	99.6	20.0
Pen-R			94.0	100	100	60.9	84.8	100	100	13.1

a. Based on data from patients with CABP and *S. pneumoniae* at baseline in the microITT population of the phase 3 OPTIC study (1). NA indicates the absence of data at a given MIC value.
b. Assessed using total-drug ELF and free-drug plasma AUC/MIC ratio targets associated with a 1-$\log_{10}$ CFU reduction from baseline for *S. pneumoniae* based on data from a neutropenic murine lung-infection model (2).
c. Based on the assessment of average 24-h total-drug ELF and free-drug plasma AUC values on days 1 and 2
d. Using data for all *S. pneumoniae* isolates studied, total-drug ELF and free-drug plasma AUC/MIC ratio targets associated with a 1-$\log_{10}$ CFU reduction from baseline were randomly assigned based on an estimated log normal distributions of AUC/MIC ratio targets associated with the same endpoint.
e. Based on data for all *S. pneumoniae* isolates studied, the median total-drug ELF and free-drug plasma AUC/MIC ratio targets associated with a 1-$\log_{10}$ CFU reduction from baseline were 13.3 and 15.2, respectively (2).
f. Based on data for all *S. pneumoniae* isolates studied, the second highest total-drug ELF and free-drug plasma AUC/MIC ratio targets associated with a 1-$\log_{10}$ CFU reduction from baseline were 17.6 and 19.7, respectively (2).
g. Based on data for all *S. pneumoniae* isolates studied, the highest total-drug ELF and free-drug plasma AUC/MIC ratio targets associated with a 1-$\log_{10}$ CFU reduction from baseline were 200.6 and 180.0, respectively (2).
h. "Overall" represents the percentage of successful clinical response among all observed patients or the percent probability of PK-PD target attainment weighted over the given MIC distribution (3) for simulated patients.

Table S2. Comparison of observed percentages of successful clinical response by MIC among patients with CABP and *S. pneumoniae* at baseline and percent probabilities of PK-PD target attainment by MIC on days 1 to 2 based on total-drug ELF and free-drug plasma AUC/MIC ratio targets associated with a 1-log_{10} CFU reduction from baseline for *S. pneumoniae*, excluding the outlier, among simulated patients after the administration of omadacycline 100 mg i.v. q12h on day 1 followed by 100 mg i.v. q24h on day 2 and 300 mg p.o. q24h on days 3 to 5

MIC (µg/mL)	% of successful clinical response by MIC (no./total) for 28 patients[a]		Percent probability of PK-PD target attainment by MIC on days 1 and 2 among simulated patients[b,c]					
			Assessment of total-drug ELF exposures and AUC/MIC ratio targets			Assessment of free-drug plasma exposures and AUC/MIC ratio targets		
	ECR at 72 to 120 hours	Clinical success at PTE	Randomly assigned based on PK-PD targets[d]	Median/second highest of PK-PD targets[e]	Highest PK-PD target[f]	Randomly assigned based on PK-PD targets[d]	Median/second highest of PK-PD targets[e]	Highest PK-PD target[f]
0.015	100 (2/2)	100 (2/2)	100	100	100	100	100	100
0.03	78.6 (11/14)	85.7 (12/14)	100	100	100	100	100	100
0.06	80.0 (8/10)	100 (10/10)	100	100	100	100	100	100
0.12	50.0 (1/2)	50 (1/2)	100	100	100	100	100	100
0.25	NA	NA	100	100	100	97.5	100	99.9
0.5	NA	NA	99.3	100	100	75.4	85.2	42.2
1	NA	NA	82.9	96.3	67.0	34.3	0.96	0.02
**Overall[g]**								
All	78.6 (22/28)	89.3 (25/28)	100	100	100	99.9	99.9	99.9
Pen-S			100	100	100	100	100	99.9
Pen-I			99.9	100	99.9	99.8	99.7	99.7
Pen-R			100	100	100	100	100	100

a. Based on data from patients with CABP and *S. pneumoniae* at baseline in the microITT population of the phase 3 OPTIC study (1). NA indicates the absence of data at a given MIC value.
b. Assessed using total-drug ELF and free-drug plasma AUC/MIC ratio targets associated with a 1-log_{10} CFU reduction from baseline for *S. pneumoniae* based on data from a neutropenic murine lung-infection model, excluding the outlier (*S. pneumoniae* 1293) (2).
c. Based on the assessment of average 24-h total-drug ELF and free-drug plasma AUC values on days 1 and 2.
d. The total-drug ELF and free-drug plasma AUC/MIC ratio targets associated with a 1-log_{10} CFU reduction from baseline were randomly assigned based on an estimated log normal distributions of AUC/MIC ratio targets associated with the same endpoint.
e. The median/second highest total-drug ELF and free-drug plasma AUC/MIC ratio targets associated with a 1-log_{10} CFU reduction from baseline were 13.3 and 15.2, respectively (2).
f. The highest total-drug ELF and free-drug plasma AUC/MIC ratio targets associated with a 1-log_{10} CFU reduction from baseline were 17.6 and 19.7, respectively (2).
g. "Overall" represents the percentage of successful clinical response among all observed patients or the percent probability of PK-PD target attainment weighted over the given MIC distribution (3) for simulated patients.

Table S3. Comparison of observed percentages of successful clinical response by MIC among patients with CABP and *S. pneumoniae* at baseline and percent probabilities of PK-PD target attainment by MIC on day 3 based on total-drug ELF and free-drug plasma AUC/MIC ratio targets associated with a 1-log_{10} CFU reduction from baseline for *S. pneumoniae*, excluding the outlier, among simulated patients after the administration of omadacycline 100 mg i.v. q12h on day 1 followed by 100 mg i.v. q24h on day 2 and 300 mg p.o. q24h on days 3 to 5

MIC (µg/mL)	% of successful clinical response by MIC (no./total) for 28 patients[a]		Percent probability of PK-PD target attainment by MIC on day 3 among simulated patients[b,c]					
			Assessment of total-drug ELF exposures and AUC/MIC ratio targets			Assessment of free-drug plasma exposures and AUC/MIC ratio targets		
	ECR at 72 to 120 hours	Clinical success at PTE	Randomly assigned based on PK-PD targets[d]	Median/second highest of PK-PD targets[e]	Highest PK-PD target[f]	Randomly assigned based on PK-PD targets[d]	Median/second highest PK-PD targets[e]	Highest PK-PD target[f]
0.015	100 (2/2)	100 (2/2)	100	100	100	100	100	100
0.03	78.6 (11/14)	85.7 (12/14)	100	100	100	100	100	100
0.06	80.0 (8/10)	100 (10/10)	100	100	100	100	100	100
0.12	50.0 (1/2)	50 (1/2)	100	100	100	99.7	100	100
0.25	NA	NA	99.9	100	100	91.0	97.9	89.5
0.5	NA	NA	95.5	99.6	95.9	59.9	44.9	17.3
1	NA	NA	67.9	66.0	31.2	22.5	1.04	0.12
**Overall[g]**								
All	78.6 (22/28)	89.3 (25/28)	100	100	99.9	99.9	99.9	99.8
Pen-S			100	100	100	99.9	99.9	99.9
Pen-I			99.9	99.9	99.8	99.6	99.7	99.6
Pen-R			100	100	100	99.9	100	100

a. Based on data from patients with CABP and *S. pneumoniae* at baseline in the microITT population of the phase 3 OPTIC study (1). NA indicates the absence of data at a given MIC value.
b. Assessed using total-drug ELF and free-drug plasma AUC/MIC ratio targets associated with a 1-log_{10} CFU reduction from baseline for *S. pneumoniae* based on data from a neutropenic murine lung-infection model, excluding the outlier isolate (*S. pneumoniae* 1293) (2).
c. Based on the assessment of average 24-h total-drug ELF and free-drug plasma AUC values on days 1 and 2.
d. The total-drug ELF and free-drug plasma AUC/MIC ratio targets associated with a 1-log_{10} CFU reduction from baseline were randomly assigned based on an estimated log normal distributions of AUC/MIC ratio targets associated with the same endpoint.
e. The median/second highest total-drug ELF and free-drug plasma AUC/MIC ratio targets associated with a 1-log_{10} CFU reduction from baseline were 13.3 and 15.2, respectively (2).
f. The highest total-drug ELF and free-drug plasma AUC/MIC ratio targets associated with a 1-log_{10} CFU reduction from baseline were 17.6 and 19.7, respectively (2).
g. "Overall" represents the percentage of successful clinical response among all observed patients or the percent probability of PK-PD target attainment weighted over the given MIC distribution (3) for simulated patients.

Table S4. Comparison of observed percentages of successful clinical response by MIC among patients with CABP and *S. pneumoniae* at baseline and percent probabilities of PK-PD target attainment by MIC on days 1 to 2 based on total-drug ELF and free-drug plasma AUC/MIC ratio targets associated with a 1-log_{10} CFU reduction from baseline for *S. pneumoniae*, including the outlier isolate, among simulated patients after the administration of omadacycline 200 mg i.v. q24h on day 1 followed by 100 mg i.v. q24h on day 2 and 300 mg p.o. q24h on days 3 to 5

MIC (µg/mL)	% of successful clinical response by MIC (no./total) for 28 patients[a]		Percent probability of PK-PD target attainment by MIC on days 1 to 2 among simulated patients[b,c]							
			Assessment of total-drug ELF exposures and AUC/MIC ratio targets				Assessment of free-drug plasma exposures and AUC/MIC ratio targets			
	ECR at 72 to 120 hours	Clinical success at PTE	Randomly assigned based on PK-PD targets[d]	Median of PK-PD targets[e]	Second highest PK-PD target[f]	Highest PK-PD target[g]	Randomly assigned based on PK-PD targets[d]	Median of PK-PD targets[e]	Second highest PK-PD target[f]	Highest PK-PD target[g]
0.015	100 (2/2)	100 (2/2)	100	100	100	100	100	100	100	100
0.03	78.6 (11/14)	85.7 (12/14)	100	100	100	100	98.5	100	100	99.7
0.06	80.0 (8/10)	100 (10/10)	98.1	100	100	99.2	92.0	100	100	31.5
0.12	50.0 (1/2)	50 (1/2)	91.4	100	100	18.7	80.6	100	100	0
0.25	NA	NA	79.8	100	100	0	63.6	100	99.9	0
0.5	NA	NA	64.4	100	100	0	44.2	71.0	48.8	0
1	NA	NA	45.8	89.2	72.8	0	26.0	0.26	0.04	0
**Overall[h]**										
All	78.6 (22/28)	89.3 (25/28)	97.7	100	100	90.9	92.1	99.9	99.9	42.5
Pen-S			98.0	100	100	93.5	92.8	100	99.9	46.0
Pen-I			97.2	100	99.9	87.4	91.1	99.7	99.7	37.3
Pen-R			96.6	100	100	79.3	89.7	100	100	28.5

a. Based on data from patients with CABP and *S. pneumoniae* at baseline in the microITT population of the phase 3 OPTIC study (1). NA indicates the absence of data at a given MIC value.
b. Assessed using total-drug ELF and free-drug plasma AUC/MIC ratio targets associated with a 1-log_{10} CFU reduction from baseline for *S. pneumoniae* based on data from a neutropenic murine lung-infection model (2).
c. Based on the assessment of average 24-h total-drug ELF and free-drug plasma AUC values on days 1 and 2.
d. Using data for all *S. pneumoniae* isolates studied, total-drug ELF and free-drug plasma AUC/MIC ratio targets associated with a 1-log_{10} CFU reduction from baseline were randomly assigned based on an estimated log normal distributions of AUC/MIC ratio targets associated with the same endpoint.
e. Based on data for all *S. pneumoniae* isolates studied, the median total-drug ELF and free-drug plasma AUC/MIC ratio targets associated with a 1-log_{10} CFU reduction from baseline were 15.5 and 17.4, respectively (2).
f. Based on data for all *S. pneumoniae* isolates studied, the second highest total-drug ELF and free-drug plasma AUC/MIC ratio targets associated with a 1-log_{10} CFU reduction from baseline were 17.6 and 19.7, respectively (2).
g. Based on data for all *S. pneumoniae* isolates studied, the highest total-drug ELF and free-drug plasma AUC/MIC ratio targets associated with a 1-log_{10} CFU reduction from baseline were 200.6 and 180.0, respectively (2).
h. "Overall" represents the percentage of successful clinical response among all observed patients or the percent probability of PK-PD target attainment weighted over the given MIC distribution (3) for simulated patients.

Table S5. Comparison of observed percentages of successful clinical response by MIC among patients with CABP and *S. pneumoniae* at baseline and percent probabilities of PK-PD target attainment by MIC on day 3 based on total-drug ELF and free-drug plasma AUC/MIC ratio targets associated with a 1-log_{10} CFU reduction from baseline for *S. pneumoniae*, including the outlier isolate, among simulated patients after the administration of omadacycline 200 mg i.v. q24h on day 1 followed by 100 mg i.v. q24h on day 2 and 300 mg p.o. q24h on days 3 to 5

MIC (µg/mL)	% of successful clinical response by MIC (no./total) for 28 patients[a]		Percent probability of PK-PD target attainment by MIC on day 3 among simulated patients[b,c]							
			Assessment of total-drug ELF exposures and AUC/MIC ratio targets				Assessment of free-drug plasma exposures and AUC/MIC ratio targets			
	ECR at 72 to 120 hours	Clinical success at PTE	Randomly assigned based on PK-PD targets[d]	Median of PK-PD targets[e]	Second highest PK-PD target[f]	Highest PK-PD target[g]	Randomly assigned based on PK-PD targets[d]	Median of PK-PD targets[e]	Second highest PK-PD target[f]	Highest PK-PD target[g]
0.015	100 (2/2)	100 (2/2)	99.9	100	100	100	99.5	100	100	99.7
0.03	78.6 (11/14)	85.7 (12/14)	99.4	100	100	99.7	95.6	100	100	77.8
0.06	80.0 (8/10)	100 (10/10)	95.3	100	100	70.4	86.7	100	100	7.98
0.12	50.0 (1/2)	50 (1/2)	86.7	100	100	4.72	73.0	100	100	0.04
0.25	NA	NA	72.8	100	100	0	53.6	92.5	85.7	0
0.5	NA	NA	55.2	97.4	93.7	0	34.9	23.6	13.7	0
1	NA	NA	37.3	40.4	25.9	0	19.1	0.22	0.10	0
**Overall[h]**										
All	78.6 (22/28)	89.3 (25/28)	95.2	100	99.9	69.7	87.1	99.9	99.8	22.1
Pen-S			95.6	100	100	72.9	88.0	99.9	99.9	25.0
Pen-I			94.4	99.8	99.8	65.3	85.8	99.6	99.6	17.5
Pen-R			93.4	100	100	56.2	84.0	100	100	11.1

a. Based on data from patients with CABP and *S. pneumoniae* at baseline in the microITT population of the phase 3 OPTIC study (1). NA indicates the absence of data at a given MIC value.
b. Assessed using total-drug ELF and free-drug plasma AUC/MIC ratio targets associated with a 1-log_{10} CFU reduction from baseline for *S. pneumoniae* based on data from a neutropenic murine lung-infection model (2).
c. Based on the assessment of average 24-h total-drug ELF or free-drug plasma AUC values on days 1 and 2.
d. Using data for all *S. pneumoniae* isolates studied, total-drug ELF and free-drug plasma AUC/MIC ratio targets associated with a 1-log_{10} CFU reduction from baseline were randomly assigned based on an estimated log normal distributions of AUC/MIC ratio targets associated with the same endpoint.
e. Based on data for all *S. pneumoniae* isolates studied, the median total-drug ELF and free-drug plasma AUC/MIC ratio targets associated with a 1-log_{10} CFU reduction from baseline were 13.3 and 15.2, respectively (2).
f. Based on data for all *S. pneumoniae* isolates studied, the second highest total-drug ELF and free-drug plasma AUC/MIC ratio targets associated with a 1-log_{10} CFU reduction from baseline were 17.6 and 19.7, respectively (2).
g. Based on data for all *S. pneumoniae* isolates studied, the highest total-drug ELF and free-drug plasma AUC/MIC ratio targets associated with a 1-log_{10} CFU reduction from baseline were 200.6 and 180.0, respectively (2).
h. "Overall" represents the percentage of successful clinical response among all observed patients or the percent probability of PK-PD target attainment weighted over the given MIC distribution (3) for simulated patients.

Table S6. Comparison of observed percentages of successful clinical response by MIC among patients with CABP and *S. pneumoniae* at baseline and percent probabilities of PK-PD target attainment by MIC on days 1 to 2 based on total-drug ELF and free-drug plasma AUC/MIC ratio targets associated with a 1-log_{10} CFU reduction from baseline for *S. pneumoniae*, excluding the outlier isolate, among simulated patients after the administration of omadacycline 200 mg i.v. q24h on day 1 followed by 100 mg i.v. q24h on day 2 and 300 mg p.o. q24h on days 3 to 5

MIC (µg/mL)	% of successful clinical response by MIC (no./total) for 28 patients[a]		Percent probability of PK-PD target attainment by MIC on days 1 to 2 among simulated patients[b,c]					
			Assessment of total-drug ELF exposures and AUC/MIC ratio targets			Assessment of free-drug plasma exposures and AUC/MIC ratio targets		
	ECR at 72 to 120 hours	Clinical success at PTE	Randomly assigned based on PK-PD targets[d]	Median/second highest of PK-PD targets[e]	Highest PK-PD target[f]	Randomly assigned based on PK-PD targets[d]	Median/second highest of PK-PD targets[e]	Highest PK-PD target[f]
0.015	100 (2/2)	100 (2/2)	100	100	100	100	100	100
0.03	78.6 (11/14)	85.7 (12/14)	100	100	100	100	100	100
0.06	80.0 (8/10)	100 (10/10)	100	100	100	100	100	100
0.12	50.0 (1/2)	50 (1/2)	100	100	100	100	100	100
0.25	NA	NA	100	100	100	98.0	100	99.9
0.5	NA	NA	99.5	100	100	77.2	89.0	48.8
1	NA	NA	84.5	97.5	72.8	36.4	1.56	0.04
**Overall**[g]								
All	78.6 (22/28)	89.3 (25/28)	100	100	100	99.9	99.9	99.9
Pen-S			100	100	100	100	100	99.9
Pen-I			99.9	100	99.9	99.8	99.7	99.7
Pen-R			100	100	100	100	100	100

a. Based on data from patients with CABP and *S. pneumoniae* at baseline in the microITT population of the phase 3 OPTIC study (1). NA indicates the absence of data at a given MIC value.
b. Assessed using total-drug ELF and free-drug plasma AUC/MIC ratio targets associated with a 1-log_{10} CFU reduction from baseline for *S. pneumoniae* based on data from a neutropenic murine lung-infection model, excluding the outlier isolate (*S. pneumoniae* 1293) (2).
c. Based on the assessment of average 24-h total-drug ELF and free-drug plasma AUC values on days 1 and 2.
d. The total-drug ELF and free-drug plasma AUC/MIC ratio targets associated with a 1-log_{10} CFU reduction from baseline were randomly assigned based on an estimated log normal distributions of AUC/MIC ratio targets associated with the same endpoint.
e. The median/second highest total-drug ELF and free-drug plasma AUC/MIC ratio targets associated with a 1-log_{10} CFU reduction from baseline were 15.5 and 17.4, respectively (2).
f. The highest total-drug ELF and free-drug plasma AUC/MIC ratio targets associated with a 1-log_{10} CFU reduction from baseline were 17.6 and 19.7, respectively (2).
g. "Overall" represents the percentage of successful clinical response among all observed patients or the percent probability of PK-PD target attainment weighted over the given MIC distribution (3) for simulated patients.

Table S7. Comparison of observed percentages of successful clinical response by MIC among patients with CABP and *S. pneumoniae* at baseline and percent probabilities of PK-PD target attainment by MIC on day 3 based on total-drug ELF and free-drug plasma AUC/MIC ratio targets associated with a 1-log_{10} CFU reduction from baseline for *S. pneumoniae*, excluding the outlier isolate, among simulated patients after the administration of omadacycline 200 mg i.v. q24h on day 1 followed by 100 mg i.v. q24h on day 2 and 300 mg p.o. q24h on days 3 to 5

MIC (µg/mL)	% of successful clinical response by MIC (no./total) for 28 patients[a]		Percent probability of PK-PD target attainment by MIC on day 3 among simulated patients[b,c]					
			Assessment of total-drug ELF exposures and AUC/MIC ratio targets			Assessment of free-drug plasma exposures and AUC/MIC ratio targets		
	ECR at 72 to 120 hours	Clinical success at PTE	Randomly assigned based on PK-PD targets[d]	Median/second highest of PK-PD targets[e]	Highest PK-PD target[f]	Randomly assigned based on PK-PD targets[d]	Median/second highest of PK-PD targets[e]	Highest PK-PD target[f]
0.015	100 (2/2)	100 (2/2)	100	100	100	100	100	100
0.03	78.6 (11/14)	85.7 (12/14)	100	100	100	100	100	100
0.06	80.0 (8/10)	100 (10/10)	100	100	100	100	100	100
0.12	50.0 (1/2)	50 (1/2)	100	100	100	99.6	100	100
0.25	NA	NA	99.9	100	100	89.0	96.8	85.7
0.5	NA	NA	94.6	99.1	93.7	56.6	38.5	13.7
1	NA	NA	64.8	59.6	25.9	20.1	0.54	0.10
**Overall[g]**								
All	78.6 (22/28)	89.3 (25/28)	100	100	99.9	99.9	99.9	99.8
Pen-S			100	100	100	99.9	99.9	99.9
Pen-I			99.9	99.9	99.8	99.6	99.6	99.6
Pen-R			100	100	100	99.9	100	100

a. Based on data from patients with CABP and *S. pneumoniae* at baseline in the microITT population of the phase 3 OPTIC study (1). NA indicates the absence of data at a given MIC value.
b. Assessed using total-drug ELF and free-drug plasma AUC/MIC ratio targets associated with a 1-log_{10} CFU reduction from baseline for *S. pneumoniae* based on data from a neutropenic murine lung-infection model, excluding the outlier isolate (*S. pneumoniae* 1293) (2).
c. Based on the assessment of average 24-h total-drug ELF and free-drug plasma AUC values on days 1 and 2.
d. The total-drug ELF and free-drug plasma AUC/MIC ratio targets associated with a 1-log_{10} CFU reduction from baseline were randomly assigned based on an estimated log normal distributions of AUC/MIC ratio targets associated with the same endpoint.
e. The median/second highest total-drug ELF and free-drug plasma AUC/MIC ratio targets associated with a 1-log_{10} CFU reduction from baseline were 13.3 and 15.2, respectively (2).
f. The highest total-drug ELF and free-drug plasma AUC/MIC ratio targets associated with a 1-log_{10} CFU reduction from baseline were 17.6 and 19.7, respectively (2).
g. "Overall" represents the percentage of successful clinical response among all observed patients or the percent probability of PK-PD target attainment weighted over the given MIC distribution (3) for simulated patients.

Table S8. Comparison of observed percentages of successful clinical response by MIC among patients with CABP and *H. influenzae* at baseline and percent probabilities of PK-PD target attainment by MIC on day 3 based on total-drug ELF and free-drug plasma AUC/MIC ratio targets associated with a 1-$\log_{10}$ CFU reduction from baseline for *H. influenzae* among simulated patients after the administration of omadacycline 100 mg i.v. q12h on day 1 followed by 100 mg i.v. q24h on day 2 and 300 mg p.o. q24h on days 3 to 5

MIC (µg/mL)	% of successful clinical response by MIC (no./total) for 28 patients[a]		Percent probability of PK-PD target attainment by MIC on day 3 among simulated patients[b,c]							
			Assessment of total-drug ELF exposures and AUC/MIC ratio targets				Assessment of free-drug plasma exposures and AUC/MIC ratio targets			
	ECR at 72 to 120 hours	Clinical success at PTE	Randomly assigned based on PK-PD targets[d]	Median of PK-PD targets[e]	Second highest PK-PD target[f]	Highest PK-PD target[g]	Randomly assigned based on PK-PD targets[d]	Median of PK-PD targets[e]	Second highest PK-PD target[f]	Highest PK-PD target[g]
0.12	NA	NA	100	100	100	100	100	100	100	100
0.25	NA	NA	100	100	100	100	99.9	100	100	99.7
0.5	100 (1/1)	100 (1/1)	99.9	100	100	99.9	91.6	94.1	90.2	76.4
1	77.8 (14/18)	88.9 (16/18)	93.0	95.6	92.3	80.3	42.0	26.4	18.3	6.76
2	50.0 (6/12)	66.7 (8/12)	45.3	29.9	21.4	8.16	3.52	0.24	0.14	0
4	100 (1/1)	100 (1/1)	4.30	0.26	0.20	0.04	0.04	0	0	0
**Overall[h]**										
All	68.8 (22/32)	81.3 (26/32)	91.3	91.2	89.0	82.4	61.1	54.9	49.5	38.4
BL-Neg			91.3	91.1	88.8	82.3	61.4	55.4	50.2	39.0
BL-Pos			91.5	91.6	89.4	82.5	60.1	53.3	47.7	36.5

a. Based on data from patients with CABP and *H. influenzae* at baseline in the microITT population of the phase 3 OPTIC study (1). NA indicates the absence of data at a given MIC value.
b. Assessed using total-drug ELF/free-drug plasma AUC/MIC ratio targets associated with a 1-$\log_{10}$ CFU reduction from baseline for *H. influenzae* based on data from one-compartment *in vitro* infection model (4).
c. Based on the assessment of average 24-h total-drug ELF and free-drug plasma AUC values on days 1 and 2.
d. Using data for all *H. influenzae* isolates studied, total-drug ELF/free-drug plasma AUC/MIC ratio targets associated with a 1-$\log_{10}$ CFU reduction from baseline were randomly assigned based on an estimated log normal distributions of AUC/MIC ratio targets associated with the same endpoint.
e. Based on data for all *H. influenzae* isolates studied, the median total-drug ELF/free-drug plasma AUC/MIC ratio target associated with a 1-$\log_{10}$ CFU reduction from baseline was 8.91 (4).
f. Based on data for all *H. influenzae* isolates studied, the second highest total-drug ELF/free-drug plasma AUC/MIC ratio target associated with a 1-$\log_{10}$ CFU reduction from baseline was 9.73 (4).
g. Based on data for all *H. influenzae* isolates studied, the highest total-drug ELF/free-drug plasma AUC/MIC ratio target associated with a 1-$\log_{10}$ CFU reduction from baseline was 11.6 (4).
h. "Overall" represents the percentage of successful clinical response among all observed patients or the percent probability of PK-PD target attainment weighted over the given MIC distribution (3) for simulated patients.

Table S9. Comparison of observed percentages of successful clinical response by MIC among patients with CABP and *H. influenzae* at baseline and percent probabilities of PK-PD target attainment by MIC on days 1 to 2 based on total-drug ELF and free-drug plasma AUC/MIC ratio targets associated with a 1-$\log_{10}$ CFU reduction from baseline for *H. influenzae* among simulated patients after the administration of omadacycline 200 mg i.v. q24h on Day 1 followed by 100 mg i.v. q24h on day 2 and 300 mg p.o. q24h on days 3 to 5

MIC (µg/mL)	% of successful clinical response by MIC (no./total) for 32 patients[a]		Percent probability of PK-PD target attainment by MIC on days 1 to 2 among simulated patients[b,c]							
			Assessment of total-drug ELF exposures and AUC/MIC ratio targets				Assessment of free-drug plasma exposures and AUC/MIC ratio targets			
	ECR at 72 to 120 hours	Clinical success at PTE	Randomly assigned based on PK-PD targets[d]	Median of PK-PD targets[e]	Second highest PK-PD target[f]	Highest PK-PD target[g]	Randomly assigned based on PK-PD targets[d]	Median of PK-PD targets[e]	Second highest PK-PD target[f]	Highest PK-PD target[g]
0.12	NA	NA	100	100	100	100	100	100	100	100
0.25	NA	NA	100	100	100	100	100	100	100	100
0.5	100 (1/1)	100 (1/1)	100	100	100	100	99.6	100	99.9	99.3
1	77.8 (14/18)	88.9 (16/18)	99.6	100	99.9	99.4	69.9	66.9	50.9	20.2
2	50.0 (6/12)	66.7 (8/12)	71.9	70.8	55.3	23.8	8.90	0.22	0.04	0
4	100 (1/1)	100 (1/1)	10.2	0.26	0.08	0	0	0	0	0
**Overall[h]**										
All	68.8 (22/32)	81.3 (26/32)	96.8	96.8	95.4	92.4	77.6	75.7	68.4	54.4
BL-Neg			96.8	96.8	95.3	92.2	77.7	75.8	68.7	55.0
BL-Pos			96.8	96.8	95.6	93.0	77.4	75.4	67.6	52.4

a. Based on data from patients with CABP and *H. influenzae* at baseline in the microITT population of the phase 3 OPTIC study (1). NA indicates the absence of data at a given MIC value.
b. Assessed using total-drug ELF/free-drug plasma AUC/MIC ratio targets associated with a 1-$\log_{10}$ CFU reduction from baseline for *H. influenzae* based on data from a one-compartment *in vitro* infection model (4]).
c. Based on the assessment of average 24-h total-drug ELF and free-drug plasma AUC on days 1 and 2.
d. Using data for all *H. influenzae* isolates, total-drug ELF/free-drug plasma AUC/MIC ratio targets associated with a 1-$\log_{10}$ CFU reduction from baseline were randomly assigned based on an estimated log normal distributions of AUC/MIC ratio targets associated with the same endpoint.
e. Based on data for all *H. influenzae* isolates, the median total-drug ELF/free-drug plasma AUC/MIC ratio target associated with a 1-$\log_{10}$ CFU reduction from baseline was 8.91 (4).
f. Based on data for all *H. influenzae* isolates studied, the second highest total-drug ELF/free-drug plasma AUC/MIC ratio target associated with a 1-$\log_{10}$ CFU reduction from baseline was 9.73 (4).
g. Based on data for all *H. influenzae* isolates studied, the highest total-drug ELF/free-drug plasma AUC/MIC ratio target associated with a 1-$\log_{10}$ CFU reduction from baseline was 11.6 (4).
h. “Overall” represents the percentage of successful clinical response among all observed patients or the percent probability of PK-PD target attainment weighted over the given MIC distribution (3) for simulated patients.

Table S10. Comparison of observed percentages of successful clinical response by MIC among patients with CABP and *H. influenzae* at baseline and percent probabilities of PK-PD target attainment by MIC on day 3 based on total-drug ELF and free-drug plasma AUC/MIC ratio targets associated with a 1-$\log_{10}$ CFU reduction from baseline for *H. influenzae* among simulated patients after the administration of omadacycline 200 mg i.v. q24h on day 1 followed by 100 mg i.v. q24h on day 2 and 300 mg p.o. q24h on days 3 to 5

MIC (µg/mL)	% of successful clinical response by MIC (no./total) for 32 patients[a]		Percent probability of PK-PD target attainment by MIC on day 3 among simulated patients[b,c]							
			Assessment of total-drug ELF exposures and AUC/MIC ratio targets				Assessment of free-drug plasma exposures and AUC/MIC ratio targets			
	ECR at 72 to 120 hours	Clinical success at PTE	Randomly assigned based on PK-PD targets[d]	Median of PK-PD targets[e]	Second highest PK-PD target[f]	Highest PK-PD target[g]	Randomly assigned based on PK-PD targets[d]	Median of PK-PD targets[e]	Second highest PK-PD target[f]	Highest PK-PD target[g]
0.12	NA	NA	100	100	100	100	100	100	100	100
0.25	NA	NA	100	100	100	100	99.8	100	100	99.6
0.5	100 (1/1)	100 (1/1)	99.9	100	100	99.7	89.4	91.7	86.4	70.4
1	77.8 (14/18)	88.9 (16/18)	90.9	93.3	88.9	74.7	38.0	21.8	14.3	5.00
2	50.0 (6/12)	66.7 (8/12)	40.4	24.8	16.7	6.18	2.80	0.22	0.12	0
4	100 (1/1)	100 (1/1)	3.40	0.22	0.12	0.02	0	0	0	0
**Overall[h]**										
All	68.8 (22/32)	81.3 (26/32)	90.0	89.7	87.0	79.6	58.3	51.8	46.1	35.0
BL-Neg			90.0	89.6	86.9	79.6	58.7	52.4	46.7	35.6
BL-Pos			90.1	90.1	87.4	79.5	57.2	50.0	44.2	33.1

a. Based on data from patients with CABP and *H. influenzae* at baseline in the microITT population of the phase 3 OPTIC study (1). NA indicates the absence of data at a given MIC value.
b. Assessed using total-drug ELF/free-drug plasma AUC/MIC ratio targets associated with a 1-$\log_{10}$ CFU reduction from baseline for *H. influenzae* based on data from a one-compartment *in vitro* infection model (4).
c. Based on the assessment of average 24-h total-drug ELF and free-drug plasma AUC values on days 1 and 2.
d. Using data for all *H. influenzae* isolates studied, total-drug ELF/free-drug plasma AUC/MIC ratio targets associated with a 1-$\log_{10}$ CFU reduction from baseline were randomly assigned based on an estimated log normal distributions of AUC/MIC ratio targets associated with the same endpoint.
e. Based on data for all *H. influenzae* isolates studied, the median total-drug ELF and free-drug plasma AUC/MIC ratio target associated with a 1-$\log_{10}$ CFU reduction from baseline was 8.91 (4).
f. Based on data for all *H. influenzae* isolates studied, the second highest total-drug ELF and free-drug plasma AUC/MIC ratio target associated with a 1-$\log_{10}$ CFU reduction from baseline was 9.73 (4).
g. Based on data for all *H. influenzae* isolates studied, the highest total-drug ELF and free-drug plasma AUC/MIC ratio target associated with a 1-$\log_{10}$ CFU reduction from baseline was 11.6 (4).
h. "Overall" represents the percentage of successful clinical response among all observed patients or the percent probability of PK-PD target attainment weighted over the given MIC distribution ([3) for simulated patients.

Table S11. Omadacycline total-drug ELF and free-drug plasma AUC/MIC ratio targets for *S. pneumoniae* efficacy based on data from a neutropenic murine-lung infection model (2)

Matrix	*S. pneumoniae* isolate	MIC value (µg/mL)	AUC/MIC ratio targets by efficacy endpoint		
			Net bacterial stasis	1-log_{10} CFU reduction from baseline	2-log_{10} CFU reduction from baseline
Total-drug ELF	1293	0.06	17.8	200.6	-
	10813	0.06	14.2	17.6	23.2
	140	0.125	-	6.00	17.3
	49619	0.03	-	13.3	47.3
	Mean (SD)	-	16.0 (2.56)	59.4 (94.3)	-
	Mean without 1293 (SD)	-	-	12.3 (5.86)	29.3 (15.9)
	Median	-	-	15.5	-
	Median without 1293	-	-	13.3	23.2
Free-drug plasma	1293	0.06	19.8	180.0	-
	10813	0.06	15.8	19.7	25.1
	140	0.125	-	6.06	18.6
	49619	0.03	-	15.2	56.2
	Mean (SD)	-	17.8 (2.86)	55.2 (83.4)	-
	Mean without 1293 (SD)	-	-	13.6 (6.93)	33.3 (20.1)
	Median	-	-	17.4	-
	Median without 1293	-	-	15.2	25.1

Table S12. Omadacycline total-drug ELF/free-drug plasma[a] AUC/MIC ratio targets for *H. influenzae* efficacy based on data from a one-compartment *in vitro* infection model (4)

H. influenzae **isolate**	**MIC (µg/mL)**	**Total-drug ELF/free-drug plasma[a] AUC/MIC ratio targets by efficacy endpoint**		
		**Net bacterial stasis**	**1-$\log_{10}$ CFU reduction from baseline**	**2-$\log_{10}$ CFU reduction from baseline**
437	1	6.91	8.91	11.1
10929	1	7.09	9.73	12.9
2696	2	4.38	5.44	6.72
49247	2	8.76	11.6	15.5
543	2	4.45	5.78	7.45
Mean (SD)	–	6.32 (1.88)	8.30 (2.64)	10.7 (3.69)
Median	–	6.91	8.91	11.1

a. Total-drug ELF and free-drug plasma AUC/MIC ratio targets for *H. influenzae* were considered to be equivalent based on the assumption that any differences in the time-course of the plasma and ELF omadacycline exposures did not impact the magnitude of the AUC/MIC ratio necessary for efficacy.

Figure S1. Percent probabilities of PK-PD target attainment by MIC on days 1 to 2 based on the evaluation of the total-drug ELF and free-drug plasma AUC/MIC ratio targets associated with a 1-log_{10} CFU reduction from baseline for *S. pneumoniae*, including and excluding the outlier, among simulated patients after the administration of omadacycline 100 mg i.v.q12h on day 1 followed by 100 mg i.v. q24h on day 2 and 300 mg p.o. q24h on days 3 to 5 (A and B, respectively), and 200 mg i.v. q24h on day 1 followed by 100 mg i.v. q24h on day 2 and 300 mg p.o. q24h on days 3 to 5 (C and D, respectively), overlaid on the MIC distribution for *S. pneumoniae*.

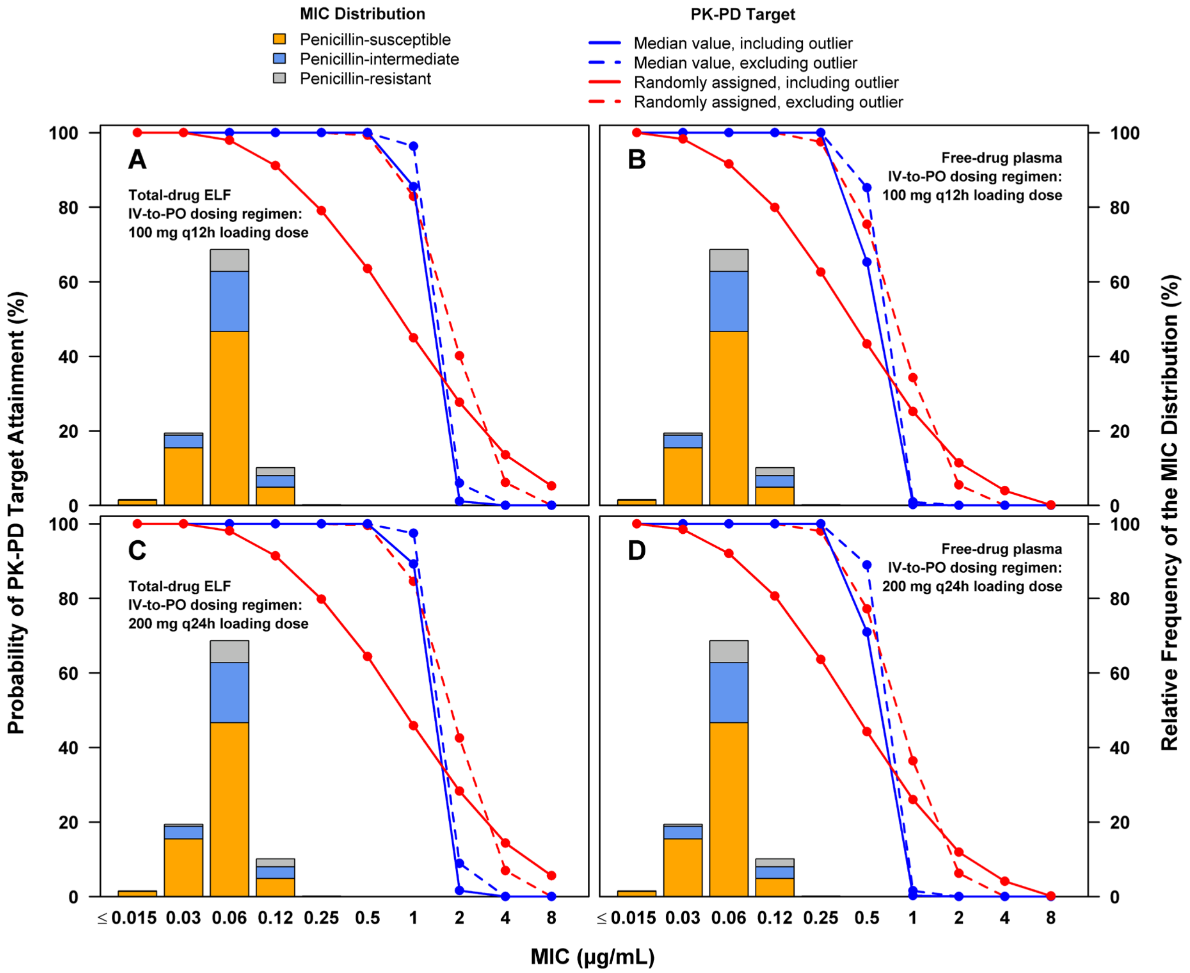

Figure S2. Non-clinical PK-PD relationship for efficacy for *S. pneumoniae*, overlaid with box-and-whisker plots of total-drug ELF and free-drug plasma AUC/MIC ratios on days 1 to 2 for simulated patients after the administration of omadacycline 100 mg i.v. q12h on day 1 followed by 100 mg i.v. q24h on day 2 and 300 mg p.o. q24h on days 3 to 5 (A and B, respectively), and 200 mg i.v. q24h on day 1 followed by 100 mg i.v. q24h on day 2 and 300 mg p.o. q24h on days 3 to 5 (C and D, respectively). Horizontal box-and-whisker plots of total-drug ELF and free-drug plasma AUC/MIC ratios on days 1 and 2 for simulated patients after administration of omadacycline IV-to-PO dosing regimens are shown overlaid on the PK-PD relationship based on data from a neutropenic murine-lung infection model for *S. pneumoniae*.

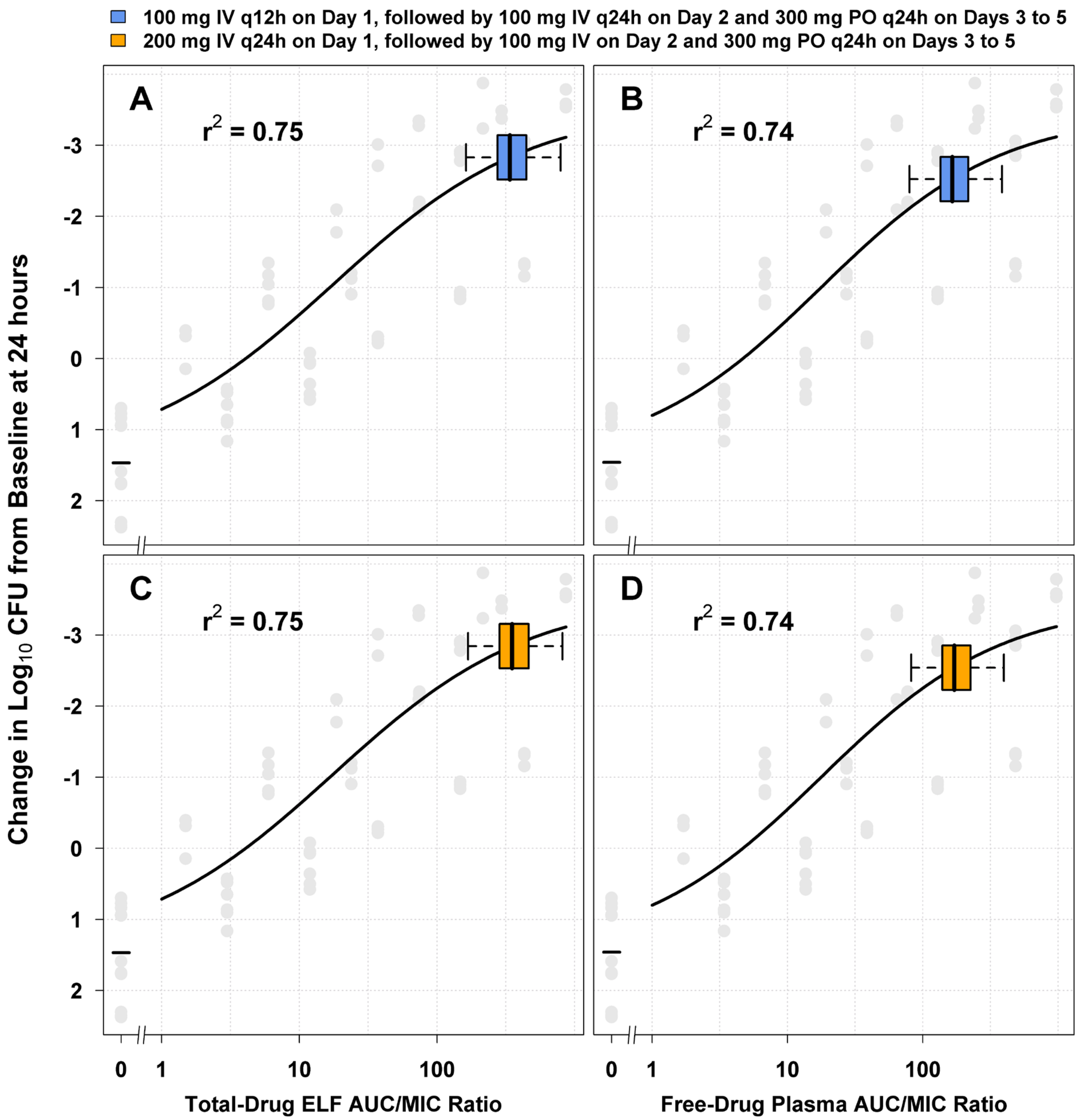

.

For each boxplot, the edge of the box represents the 25th to the 75th percentiles of the distribution for total-drug ELF or free-drug plasma AUC/MIC ratio. The line within the box represents with median total-drug ELF or free-drug plasma AUC/MIC ratio. The whiskers extend to the nearest value among those represented by 1.5 × IQR of the box edges, where IQR is interquartile range as defined by the distribution of total-drug ELF or free-drug plasma AUC/MIC ratio from the 25th to the 75th percentiles.

Figure S3. Percent probabilities of PK-PD target attainment by MIC on days 1 to 2 based on the evaluation of the total-drug ELF and free-drug plasma AUC/MIC ratio targets associated with a 1-log_{10} CFU reduction from baseline for *H. influenzae* among simulated patients after the administration of omadacycline 100 mg i.v. q12h on day 1 followed by 100 mg i.v. q24h on day 2 and 300 mg p.o. q24h on days 3 to 5 (A and B, respectively), and 200 mg i.v. q24h on day 1, followed by 100 mg i.v. q24h on Day 2 and 300 mg p.o. q24h on days 3 to 5 (C and D, respectively), overlaid on the MIC distribution for *H. influenzae*.

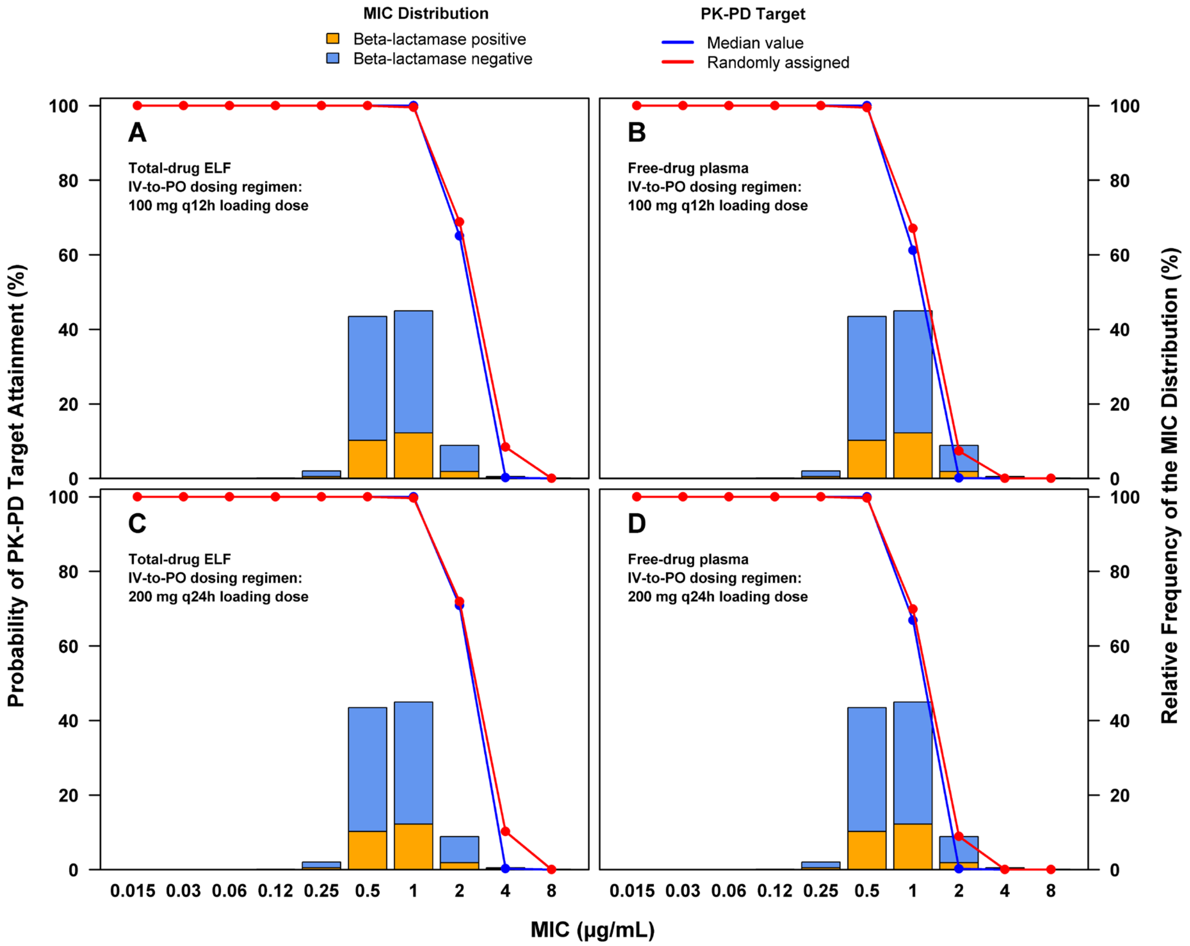

Figure S4. Non-clinical PK-PD relationship for efficacy for *H. influenzae*, overlaid with box-and-whisker plots of total-drug ELF and free-drug plasma AUC/MIC ratios on days 1 to 2 for simulated patients after administration of omadacycline 100 mg i.v. q12h on day 1 followed by 100 mg i.v. q24h on day 2 and 300 mg p.o. q24h on days 3 to 5 (A and B, respectively) and 200 mg i.v. q24h on day 1 followed by 100 mg i.v. q24h on day 2 and 300 mg p.o. q24h on days 3 to 5 (C and D, respectively). Horizontal box-and-whisker plots of total-drug ELF and free-drug plasma AUC/MIC ratios on days 1 and 2 for simulated patients after administration of omadacycline IV-to-PO dosing regimens are shown overlaid on the PK-PD relationship based on data from a one-compartment *in vitro* infection model for *H. influenzae*.

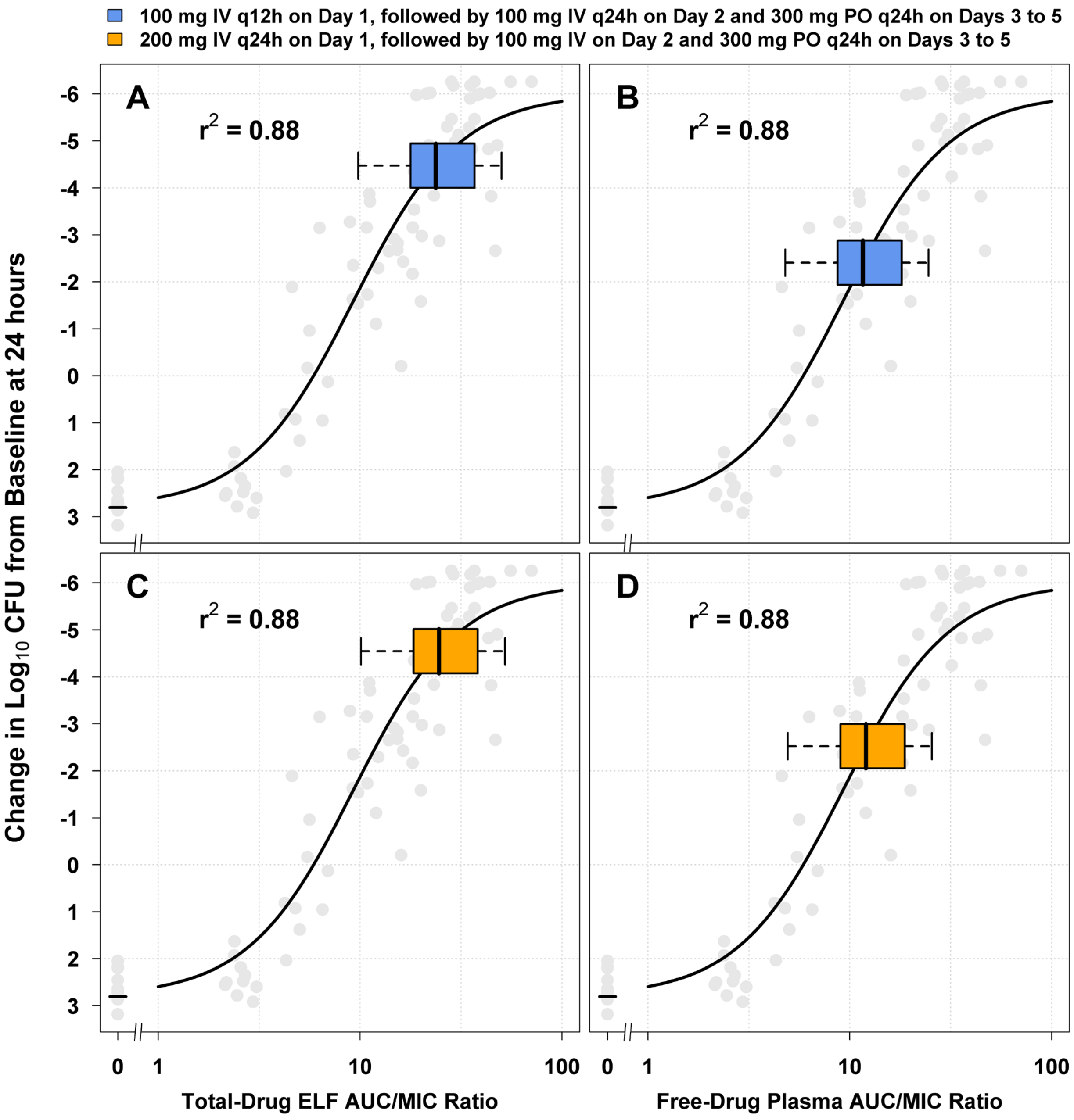

For each boxplot, the edge of the box represents the 25th to the 75th percentiles of the distribution for total-drug ELF or free-drug plasma AUC/MIC ratio. The line within the box represents with median total-drug ELF or free-drug plasma AUC/MIC ratio. The whiskers extend to the nearest value among those represented by 1.5 × IQR of the box edges.

Figure S5. Truncated log-normal distributions estimated using individual free-drug plasma AUC/MIC ratio targets for *S. pneumoniae* obtained from the neutropenic murine lung infection model associated with a 1-log_{10} CFU reduction from baseline, including and excluding the outlying highest individual plasma AUC/MIC ratio target

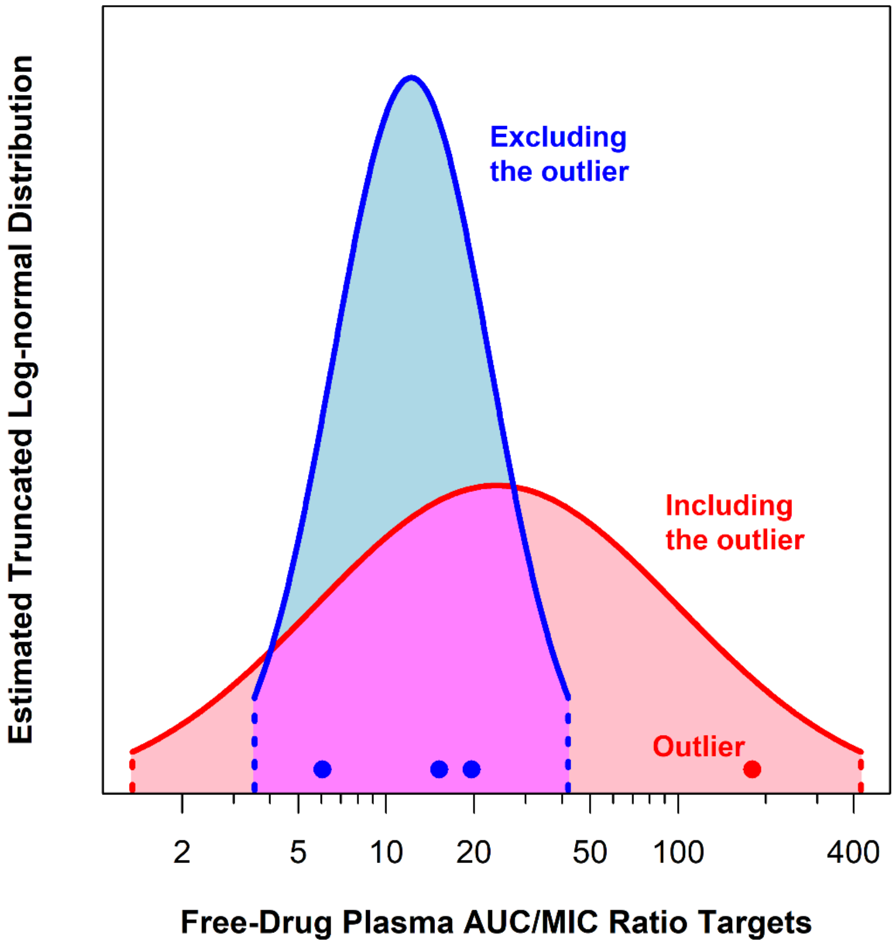

References

1. Stets R, Popescu M, Gonong JR, Mitha I, Nseir W, Medej A, Kirsch C, Das AF, Garrity-Ryan L, Steenbergen JN, Manley A, Eckburg PB, Tzanis E, McGovern PC, Loh E. 2019. Omadacycline for community-acquired bacterial pneumonia. N Engl J Med 380:517-27.
2. Lepak AJ, Zhao M, Marchillo K, VanHecker J, Andes DR. 2017. *In vivo* pharmacodynamics evaluation of omadacycline (PTK 0796) against *Streptococcus pneumoniae* in the murine pneumonia model. Antimicrob Agents Chemother 61:e02368-16.
3. Pfaller MA, Huband MD, Shortridge D, Flamm RK. 2018. Surveillance of omadacycline activity tested against clinical isolates from the United States and Europe as part of the 2016 SENTRY antimicrobial surveillance program. Antimicrob Agents Chemother 62:e02327-17.
4. VanScoy BD, Lakota EA, Conde H, McCauley J, Friedrich L, Steenbergen JN, Ambrose PG, Bhavnani SM. 2018. Pharmacokinetic-pharmacodynamic characterization of omadacycline against *Haemophilus influenzae* using a one-compartment *in vitro* infection model. Antimicrob Agents Chemother 64: e02265-19. https://doi.org/10.1128/AAC.02265-19.
